# Advanced application of nanotechnology in active constituents of Traditional Chinese Medicines

**DOI:** 10.1186/s12951-023-02165-x

**Published:** 2023-11-29

**Authors:** Chong Qiu, Jun Zhe Zhang, Bo Wu, Cheng Chao Xu, Huan Huan Pang, Qing Chao Tu, Yu Qian Lu, Qiu Yan Guo, Fei Xia, Ji Gang Wang

**Affiliations:** 1 State Key Laboratory for Quality Ensurance and Sustainable Use of Dao-Di Herbs, Artemisinin Research Center, and Institute of Chinese Materia Medica, China Academy of Chinese Medical Sciences, Beijing, 100700 China; 2 Department of Traditional Chinese Medical Science, Sixth Medical Center of the Chinese PLA General Hospital, Beijing, 100037 China; 3 Department of Physiology, Yong Loo Lin School of Medicine, National University of Singapore, Singapore, 117600 Singapore

**Keywords:** Nanotechnology, Nanocarrier, Nanodrug, Traditional Chinese Medicines, Delivery

## Abstract

Traditional Chinese Medicines (TCMs) have been used for centuries for the treatment and management of various diseases. However, their effective delivery to targeted sites may be a major challenge due to their poor water solubility, low bioavailability, and potential toxicity. Nanocarriers, such as liposomes, polymeric nanoparticles, inorganic nanoparticles and organic/inorganic nanohybrids based on active constituents from TCMs have been extensively studied as a promising strategy to improve the delivery of active constituents from TCMs to achieve a higher therapeutic effect with fewer side effects compared to conventional formulations. This review summarizes the recent advances in nanocarrier-based delivery systems for various types of active constituents of TCMs, including terpenoids, polyphenols, alkaloids, flavonoids, and quinones, from different natural sources. This review covers the design and preparation of nanocarriers, their characterization, and in vitro/vivo evaluations. Additionally, this review highlights the challenges and opportunities in the field and suggests future directions for research. Nanocarrier-based delivery systems have shown great potential in improving the therapeutic efficacy of TCMs, and this review may serve as a comprehensive resource to researchers in this field.

**Graphical abstract:**

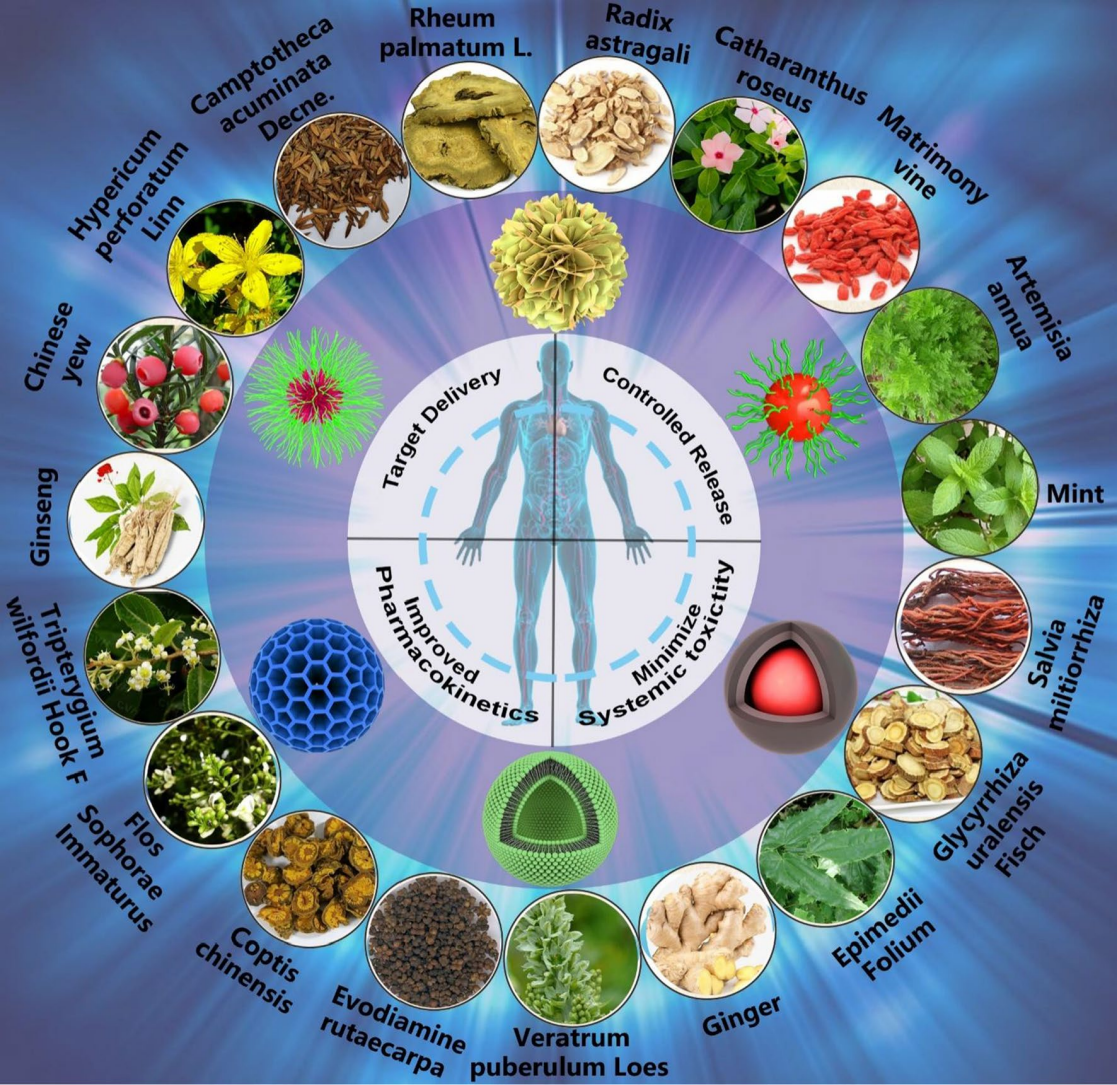

## Introduction

Traditional Chinese Medicines (TCMs) not only provide a valuable source for treating various diseases but also play a significant role in the field of medicine. The active constituents of TCMs, the vast majority of which derived from plants (about 87%), are increasingly gaining popularity for their potential health benefits and therapeutic applications [1–3]. However, many components and ingredients of TCMs face limitations such as uncontrollable quality, low solubility, poor stability, adverse effects, and inadequate targeting, hampering their extensive production and usage [4]. The therapeutic potential of TCMs has long been attributed to the functional groups present in the active ingredients of TCMs, including carboxyl, alcohol, phenol and amine, etc. These functional groups, comprising more than half of TCMs components, have been shown to possess pharmacological activity that is responsible for its unique medicinal effects, including compatibility issues, combination of multiple components, and multi-target action [5–7]. As such, a comprehensive investigation and application of these active molecules or groups hold great promise for advancing TCMs as a viable therapeutic option.

Drug delivery research using nanocarriers offers remarkable advantages for overcoming the specific shortcomings of TCMs, such as low bioavailability, poor water solubility, and unsatisfactory stability. In recent years, the number of published articles on active molecule delivery via nanocarriers has increased, reflecting a deeper understanding of their properties [8–10]. Nanocarriers can improve TCMs’ bioavailability and target action while minimizing their side effects. Passive targeting of TCMs can be achieved through nanocarriers' enhanced permeability and retention (EPR) effect, which enables them to reach target areas more efficiently [11, 12]. Active localization strategies are also possible with certain nanocarriers, which can bind to specific receptors of ligands and enhance TCMs targeting ability. Nanocarriers can prolong drug release time, maintain controllable release, and reduce toxins, maximizing therapeutic effect as well as improve the bioavailability of hydrophobic TCMs components by enhancing water solubility and stability [13–15].

**Fig. 1:**
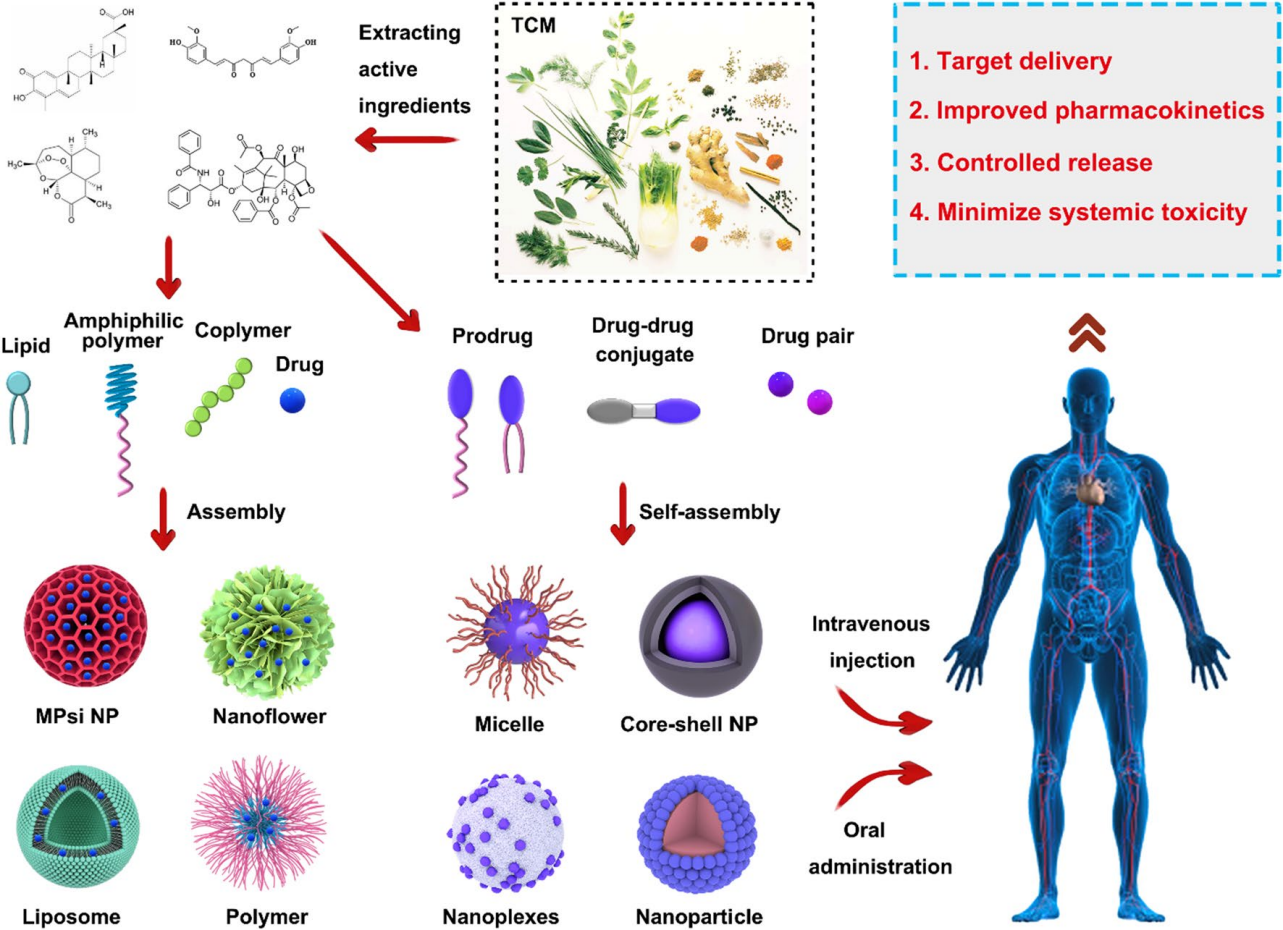
Structural illustration of applications in nanodrugs of TCMs

As shown in Fig. 1, this review analyzes the physical and chemical properties of active molecules commonly used in TCMs, such as terpenoids, polyphenols, flavonoids, alkaloids, and quinones, and summarizes the use of nanocarriers with different structural systems, including lipid-based nanocarriers, microemulsion and nanoemulsion, ethosomes and transfersomes, polymer nanocarriers, inorganic nanocarriers, and hybrid nanocarriers, discussing their advantages and limitations in TCMs drug delivery. It offers a new direction of thought for the nanodrugs delivery of TCMs.

## Common active ingredients from TCMs

### Terpenoids

**Fig. 2:**
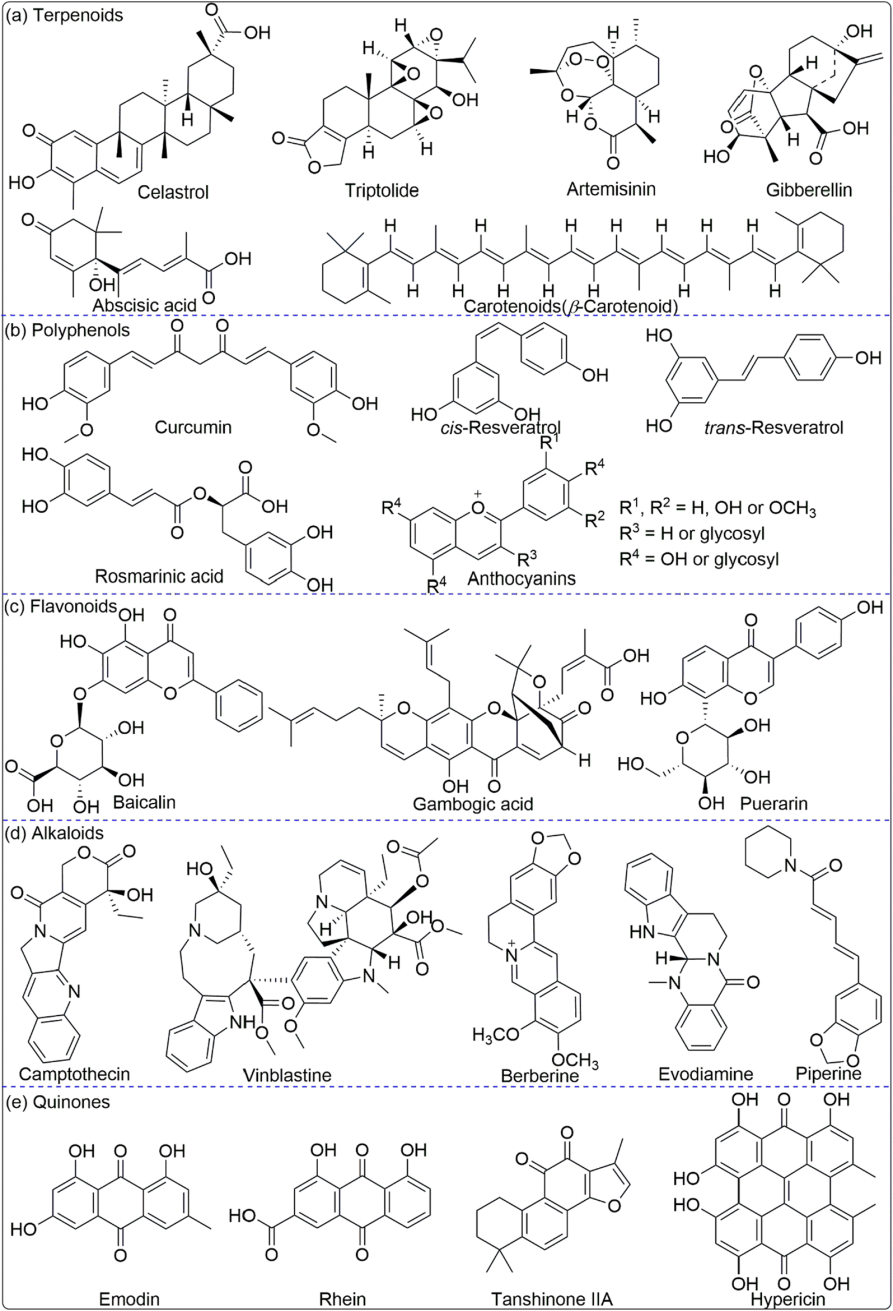
Chemical structures of active ingredients from TCMs

Terpenoids, or isoprenoids, are a large class of natural products that comprise one of the most numerous families of organic compounds in nature (e.g., triptolide, celastrol, artemisinin, Fig. 2a). These structurally diverse compounds are biosynthesized from simple building blocks, isopentyl diphosphate (IPP) and dimethylallyl diphosphate (DMAPP), through the isoprenoid pathway, a complex metabolic network composed of several enzymatic steps [16–18]. Terpenoids exist ubiquitously in the plant kingdom, but are also found in fungi, bacteria, and animals, and are well known for their diverse pharmacological activities and biological functions.

Studies have shown that terpenoids exhibit a broad range of biological activities such as anti-inflammatory, antimicrobial, anticancer, and antiparasitic properties, which have made them attractive targets for the development of novel drug candidates [19–23]. Moreover, some terpenoids, such as carotenoids, serve critical roles in photosynthesis, while others, such as gibberellins and abscisic acid, act as plant hormones that regulate a diverse array of developmental processes (Fig. 2a). Terpenoids are also involved in plant-environment interactions, facilitating the plant defense system against herbivores and pathogens, as well as providing communication signals in plant-pollinator interactions and interspecies competition [24–26].

Terpenoids exhibit remarkable structural diversity, with variations in backbone length, functional groups, and stereochemistry, which has led to a vast array of structurally complex and biologically active molecules [27–29]. In summary, the unique structurally diverse and biologically active nature of terpenoids makes them a rich source of potential therapeutic agents and a subject of extensive research in the field of natural product chemistry.

### Polyphenols

Polyphenols are a diverse group of natural compounds that are widely distributed in the plant kingdom, and are known for their potent antioxidant and anti-inflammatory properties [30, 31]. These bioactive compounds are structurally characterized by the presence of one or more phenolic rings, and are biosynthesized through the shikimate and/or polyketide pathways in plants [32–34]. Polyphenols have been extensively studied for their bioactivity and effects on human health, particularly in the prevention and treatment of chronic diseases such as cancer, cardiovascular and neurodegenerative disorders, and diabetes [35–37]. These bioactive compounds have been shown to modulate key cellular signaling pathways and enzyme activities, leading to enhanced antioxidant defense, anti-inflammatory effects, and improved metabolic function [38].

Furthermore, polyphenols exhibit remarkable structural diversity, ranging from simple phenolic acids to complex flavonoids, stilbenes, and lignans, and are present in a variety of dietary sources, including fruits, vegetables, nuts, and beverages such as tea, coffee, and wine [39–41]. As such, polyphenols have received much attention as potential therapeutic agents and nutraceuticals, and their bioavailability, metabolism, and health effects continue to be the subject of intensive research [42]. In summary, the diverse structural and bioactive properties of polyphenols make them an important class of natural compounds with potential health-promoting effects, and their exploration and utilization hold significant promise for the development of novel therapeutics and functional foods.

#### Curcumin

Curcumin (C_21_H_20_O_6_, Fig. 2b), a yellowish polyphenol obtained from the rhizome of *Curcuma longa* plants in the *Zingiberaceae* family, is used in TSMs systems to treat a range of ailments, including arthritis, stomach ulcers, dysentery, sprains, and skin infections [43–45]. It has shown significant antitumor activity in liver, stomach, and prostate cancers, and promising results as a treatment for brain diseases, cholesterol, and endothelial dysfunction [46–48]. Additionally, curcumin is a low cytotoxicity drug and an effective anti-inflammatory and antiviral agent. Curcumin can inhibit cell proliferation through proliferating cell nuclear antigen (PCNA), block the cell cycle, and induce apoptosis through mitochondrial hyperpolarization and lowered levels [49, 50]. However, curcumin’s poor water solubility and sensitivity to light and alkaline environments limit its effective concentration. To overcome these issues, curcumin nano-encapsulation via various techniques is a viable approach [51]. Nanocarriers improve solubility, increase stability, and can deliver curcumin to target sites, enhancing its biological activity while reducing toxicity.

#### Resveratrol

Resveratrol (C_14_H_12_O_3_, Fig. 2b), also known as 3,4,5-trihydroxy-trans-stilbene, was isolated from the roots of *Veratrum puberulum Loes*, and has been subsequently isolated from over 70 plant species. This natural polyphenolic substance is particularly abundant in *Veratrum puberulum Loes*, grapes, peanuts, and *Polygonum cuspidatum*. The pure product of resveratrol appears as colorless needle-like crystals, with poor solubility in water but high solubility in organic solvents such as acetone and ethanol. Resveratrol has been shown to exist in both *cis* and *trans* configuration, with the *trans*-isomer being the predominant form, while the *cis*-isomer is produced from the trans isomer under light conditions [52, 53].

Resveratrol is known to form glycosides with sugar molecules, which are subsequently cleaved by glycosidases in the intestine to release free resveratrol upon ingestion. Numerous studies have elucidated the diverse and extensive biological activities of resveratrol, which includes antioxidant, anti-inflammatory effects [54–57], anti-cancer effects (through the regulation of cell cycle and oncogenes, blockade of cancer cell pathways, and induction of autophagy) [58, 59], as well as cardiovascular protection (anti-apoptotic effect, improving the effect of cardiac hepatocyte transplantation and relieving ischemia–reperfusion injury) [60–62], neuroprotection [63, 64], osteoporosis treatment (effects on the process of cartilage aging, cartilage matrix metabolism and cartilage damage) [65, 66] and antiviral [67–69].

Despite its potential therapeutic benefits, resveratrol has poor water solubility, which is only 0.03 g/L in water leading to low oral bioavailability. Encapsulation of resveratrol in nanocarriers has demonstrated a promising solution by providing sustained release and targeted delivery properties, mitigating the drug’s side effects, and amplifying its therapeutic efficacy [70, 71]. However, the improvement of its bioavailability and large-scale production have yet to be studied in detail.

#### Rosmarinic acid

Rosmarinic acid (RA, C_18_H_16_O_8_) is a natural polyphenolic compound that is widely distributed in plants of the *Lamiaceae* and *Boraginaceae* families [72, 73]. RA was first identified and characterized in 1958 by two Italian scientists, who named it in reference to its source, *Rosmarinus officinalis*. RA’s structure comprises an ester formed via the condensation of caffeic acid and 3,4-dihydroxyphenyllactic acid, as depicted in Fig. 2b [74]. Pure RA is a white crystalline solid with a molar mass of 360.2 g/mol, a density of 1.54 g/cm^3^ and a melting point of ~ 171 °C [75]. A vast body of literature has provided compelling evidence of the diverse biological activities of RA, such as antioxidant, anti-inflammatory, antiviral, and antithrombotic effects [76–79]. RA's biological activities are attributed to its unique chemical structure, which features multiple phenolic groups, allowing it to scavenge free radicals and inhibit oxidative stress. Additionally, RA has been demonstrated to regulate pivotal signaling pathways and modulate gene expression related to inflammation, thrombosis, and viral infections, highlighting its potential as a promising therapeutic target for the development of innovative therapeutic agents.

The potential anti-inflammatory properties of RA have been investigated based on its inhibitory effects on lipoxygenases and cyclooxygenases [72]. It is reported that RA can protect the lung tissue against side effects of malathion based on its excellent anti-oxidant, anti-inflammatory and anti-apoptotic properties [80]. In vitro studies have demonstrated that RA may prevent cardiovascular disease by targeting multiple signaling pathways, including anti-myocardial fibrosis, reducing cardiac dysfunction and inhibiting myocardial apoptosis [81, 82]. In addition, the existing literature indicates that RA may hold promise as an inhibitor of cancer cell growth and inducer of apoptosis [83]. Overall, RA is a fascinating natural compound that is widely distributed in plants and has diverse biological activities. Ongoing research aims to elucidate the molecular mechanisms of its bioactivity and the development of optimized delivery systems to enhance therapeutic potential of RA.

#### Anthocyanins

Anthocyanins (ANCs), water-soluble pigments mainly found in fruits and vegetables, exhibit various colors including blue, purple, and red, and are the primary polyphenolic components in red cabbage, colored plant and vegetable extracts with inherent antioxidant activity [84, 85]. ANCs are 2-phenylbenzopyranonium cation derivatives consisting of rings A, B, and the heterocyclic C, differing in the groups attached to the B ring (Fig. 2b) [86, 87]. Over 250 naturally occurring ANCs have been identified, with commonly found in six plants: geranium, cyanidin, delphinidin, peony, morning glory, and mallow [88]. ANCs elicit a broad range of biological activities, which include anti-tumor, anti-inflammatory, antioxidant, memory-enhancing, blood pressure-reducing, cognition-enhancing, radiation-resistance-promoting, and anti-atherosclerotic effects [89–92]. Nonetheless, their vulnerability to deterioration brought about by environmental elements such as temperature, oxygen, ionic strength, gastrointestinal enzymes, acidity, and alkalinity, presents considerable obstacles [88]. Coating anthocyanins with chitosan and its derivatives has emerged as a potent approach to improve their stability against physical and oxidative deterioration, preserve their antioxidant activity, and decrease degradation rates under conditions such as simulated gastrointestinal digestion and storage [93, 94].

### Flavonoids

Flavonoids are the most prevalent secondary metabolites produced by natural selection in plants, and are found in various plant organs, particularly in petals, leaves, and fruits [95]. These compounds are abundant in many dietary sources, including soybeans, red grapes, apples, and tea [96, 97]. Flavonoids are a class of compounds that share a common basic parent nucleus of 2-phenyl chromogen ketone (C6-C3-C6), with aromatic rings of A and B rings connected in three carbon atoms [98]. Natural flavonoids could be broadly categorized into eight classes based on the degree of hybridization of the intermediate carbon atom and the bonding position of the B-ring (at position 2 or 3), including flavonoids, flavonols, dihydroflavonoids, dihydroflavonols, isoflavones, flavan-3-ols, anthocyanins, and chalcones (e.g., baicalin, gambogic acid, puerarin, Fig. 2c) [99, 100].

Flavonoids are typically crystalline granules or powders, with the color of flavonoid compounds depending on the number, location, and type of substitution of cross-conjugate systems and chromophores in the molecule. Flavonols, flavonoids, and their glycosides are typically greyish yellow to yellow, while chalcones exhibit yellow to orange–yellow hues. Isoflavones, dihydroflavonoids, and dihydroflavonols, on the other hand, are colorless due to the lack of conjugate systems [96]. Due to hydroxyl glycosidation, flavonoids tend to exhibit increased solubility in water and decreased solubility in organic solvents. Their acidic nature, arising from the presence of numerous phenolic hydroxyl groups, makes them soluble in alkaline solutions such as pyridine and formamide. Under ultraviolet (UV) light at 254 nm or 365 nm, flavonoids exhibit distinct fluorescence colors, which are further enhanced upon reaction with soda solution. Flavonoids have the ability to form colored complexes with aluminum, lead, magnesium, and other metal ions, with flavonoids typically exhibiting orange hues and most flavonols showing purple or red colors [101]. Complexation reactions are essential for the quantitative analysis of different types of flavonoids, relying on the maximum absorption wavelengths of the formed colored complexes [102].

Flavonoids have been shown to possess significant medicinal value and potential preventive effects against cardiovascular and cerebrovascular diseases [103]. These compounds can improve blood vessel strength, lower cholesterol and blood lipid levels, enhance blood vessel flow, and prevent common conditions such as cerebral hemorrhages, coronary heart disease, hypertension, and angina in the elderly [104–106]. Furthermore, many flavonoid compounds possess cough-suppressing, blood congestion-relieving, and antibacterial effects, while also exhibiting antioxidant and free radical scavenging properties [107–110]. However, flavonoids still have many obstacles in clinical use, including poor water solubility, poor oral absorption (< 5%) and fast metabolism [111], which might be solved by nanotechnology in future.

### Alkaloids

#### Camptothecin and its derivatives

A pentacyclic compound called Camptothecin (CPT, C_20_H_16_N_2_O_4_) was first discovered from by American chemists Wall and Wani in 1966 [112]. CPT consists of five rings: a pyridinone ring (also known as ring D), a pyrrole ring (A, B, and C rings), and a six-membered *α*-hydroxylactone ring (ring E) (Fig. 2d). The lactone ring is a unique structure in CPT, which has an asymmetric center and a 20S configuration with asymmetric hydroxyl groups, rendering it a potent antitumor agent [113, 114]. CPT and its derivatives are generally not soluble in water, but they can dissolve well in polar aprotic solvents such as methanol and dimethyl sulfoxide [115]. CPT solutions exhibit violet-blue fluorescence under UV, and the ring-closing lactone form has a greater impact on its biological activity [116].

CPT was first applied in mouse experiments by Wall and Wani, which surprisingly revealed its potent antitumor properties [117, 118]. Subsequently, the National Cancer Institute conducted experiments on mouse L1210 leukemia and rat Walker carcinosarcoma models, which further validated its clinical potential. However, CPT’s significant toxicity and negative effects on the urinary and digestive systems, coupled with its limited solubility and unpredictability of drug–drug interactions, have hampered further research [119, 120]. In 1985, Hsiang and his team discovered that CPT is a specific inhibitor of topoisomerase I, which rekindled interest in its potential as an antitumor drug [121–123]. Since then, topotecan (TPT) and irinotecan (CPT-11) have emerged as the main CPT derivatives used in clinical practice and have shown significant therapeutic effects [124]. Ongoing investigations are evaluating other compounds, such as 9-aminocamptothecin, GI147211, 9-nitrocamptothecin, and dx-8951f, to determine their efficacy and toxicity profiles compared to existing analogues [125].

#### Vinblastine and its derivatives

*Catharanthus roseus*, a member of the *Apocynaceae* family, is commonly cultivated for its diverse flower color, profuse blooming, and extended flowering period in warm climates. In the late 1950s, Noble et al. serendipitously discovered that a plant extract from *C. roseus* possessed a leukopenic effect in addition to its previously identified hypoglycemic properties [126]. Subsequent isolation and analysis of the extract chemicals indicated the presence of vinblastine (VLB, C_46_H_58_N_4_O_9_) and vincristine, potent antitumor agents that have been utilized in clinical practice for over six decades [127, 128]. VLB, a diterpenoid indole alkaloid with complex chemical structure (Fig. 2d) and low thermal stability, possesses needle-like white crystals that is soluble in common organic solvents such as methanol, acetone, and ethyl acetate while being insoluble in water and petroleum ether [129–131]. VLB is susceptible to decomposition due to exposure to light and therefore requires protection. In this context, the scarcity of this valuable drug is due, in part, to its challenging synthesis and isolation techniques.

VLB is characterized by myelosuppressive toxicity and is composed of the upper half of verapamil and the lower half of vindoline [132, 133]. In contrast, vincristine has a unique neurological toxicity, but little myelosuppressive toxicity and a stronger inhibitory effect on transplanted tumors due to the oxidation of the 1-*N*-methyl group on the vindoline site [134]. This small structural difference has attracted significant research interest due to its pronounced effect on the drugs’ anti-tumor and anti-toxic properties. The development of novel VLB-based antitumor agents using chemical structure modification is research-intensive, but has led to the commercialization of highly potent, low-toxicity agents such as Vindseine (VDS), Vinorebline (NVB), and Vinfulunine (VFL) [135]. Despite their effectiveness, VLB and related drugs are limited in clinical application due to their peripheral neurotoxicity, myelosuppressive toxicity, and P-glycoprotein (Pgp)-mediated resistance emerging in late clinical stages [136, 137]. Reducing the toxicity and improving the resistance of these drugs remain a significant challenge in cancer treatment.

#### Berberine

Berberine (BBR, C_20_H_18_NO_4_) is a widely studied isoquinoline (Fig. 2d) alkaloid, isolated and extracted from *Coptis chinensis*, a member of the *Ranunculaceae* plant family [138]. In nature, it is mainly found as a quaternary ammonium salt, with distribution in the roots, stems, and leaves of *Berberidaceae*, *Loganiaceae*, and *Ranunculaceae* plant families. While BBR can be synthesized in large quantities, its solubility in organic solvents remains limited, with greater solubility observed in cold water and ethanol with increasing temperature [139–141]. The yellow needle-like crystals of pure BBR have a melting point of approximately 145 °C and exhibit yellow fluorescence under UV light [142].

BBR has been extensively studied due to its diverse biological and pharmacological properties. Recent pharmacological studies have demonstrated its cardioprotective and neuroprotective effects, as well as its antibacterial and anti-inflammatory properties [138, 143–147]. Furthermore, BBR has been shown to have potent anti-diabetic and anti-tumor effects through various mechanisms. For instance, it was confirmed that BBR can suppress the inflammatory response of neutrophils by inhibiting the onset of neutrophilic respiration, thereby reducing the generation of reactive oxygen species (ROS) [148]. BBR can target key enzymes such as cyclooxygenase-2 and topoisomerase to inhibit the growth of various tumor cells [149]. Additionally, BBR has been shown to promote glucose uptake and reduce blood sugar levels by enhancing insulin sensitivity and thus protecting isolated islet cells [150–153]. Furthermore, BBR has demonstrated antiarrhythmic effects by modulating ion channels and receptors that regulate heart function. Recent studies have also highlighted the potential of BBR to reverse tumor multidrug resistance by promoting apoptosis, inhibiting drug efflux and altering the tumor microenvironment [154–157]. Together, these findings underscore the significant therapeutic potential of BBR for a range of medical applications.

#### Evodiamine

Evodiamine (EVO, C_19_H_17_N_3_O) is an indole alkaloid that originates from *Evodiamine rutaecarpa*, a traditional medicinal ingredient. It is comprised of a distinct pentacyclic backbone consisting of three nitrogen atoms (Fig. 2d) [158, 159]. Research has shown that EVO exhibits multiple pharmacological activities, including analgesia, anti-tumor, antibacterial, and metabolic disease regulation. EVO has also been found to exhibit various biological activities, e.g., antithrombotic and vasodilator activity, anti-inflammatory and anti-obesity effects, thermoregulatory and cardiovascular protection [160–163]. EVO’s wide range of activities indicates its potential for diverse therapeutic uses, such as combating cancer, inflammation, obesity, and cardiovascular disease [164–167].

#### Piperine

Piperine, the most abundant alkaloid found in peppercorns (*Piper nigrum L.*), was first isolated in 1819 by Oersted et al. [168]. It is a yellow crystalline substance belonging to the amine alkaloids of *Cinnamomum cassia*, primarily derived from the dried and ripe fruits of Piperaceae plants such as *Piper longum L* [169–172]. Piperine is the quality standard component of traditional medicine and has low water solubility (40 mg/L at 18 °C) and a molecular formula of C_17_H_19_NO_3_, as shown in Fig. 2d.

Piperine exhibits a range of pharmacological effects, including gastric ulcer treatment [173–175], hypolipidemic properties [176–178], and antioxidant activity [179–181]. Piperine can also act as an antidepressant by upregulating levels of 5-hydroxyamine or dopamine in the central nervous system [182–184], and has demonstrated analgesic [185–187] and anti-tumor effects [188–191]. Moreover, piperine has demonstrated broad-spectrum bactericidal activity with no drug resistance [192–195], and has demonstrated potential as a hypnosis and anticonvulsant agent [196–198]. The diverse range of pharmacological effects of piperine highlights its potential therapeutic applications for a variety of diseases, emphasizing the importance of further research in this area.

### Quinones

#### Emodin

Emodin (EM, C_15_H_10_O_5_) is a natural anthraquinone (Fig. 2e) derivative, mainly extracted from the rhizome of *Reynoutria japonica Houtt.* and Rh*eum palmatum L.*, and is also the main active ingredient of TMC rhubarb [199]. Pure EM is a yellow, long, needle-like crystal, with a melting point of around 256 °C, poor water solubility (70 mg/L), but soluble in ethanol and alkali solution.

Extensive research has been conducted on EM to examine its pharmacological benefits, including its ability to detoxify, combat bacterial infections, reduce inflammation, and protect the liver [200–204]. It has a weak laxative effect and is generally used as a laxative abroad [205, 206]. New research has highlighted the unforeseen antidiabetic properties of this compound, as well as its ability to impede the proliferation and viscosity of tumor cells at a cellular level and act as a cytotoxic agent against various tumors [207–210]. Moreover, EM is commonly used to inhibit bacteria, such as typhoid, urinary tract infections, and otitis media [211, 212]. Studies conducted on living organisms have revealed that EM is predominantly absorbed in the small intestine and is primarily distributed throughout the liver and kidney, aside from the metabolized portion.

EM can also be used as an attractant for fish and shrimps, a natural dye, and a fluorescent molecular probe. Solid forms of dyes, especially fluorescent dyes, can significantly impact color and fluorescence properties [213, 214]. External stimuli, such as mechanical forces, electricity, and organic solvent vapors, can induce changes in fluorescence properties by initiating the crystallization process of solid materials. Mechanical force-induced discoloration refers to the reversible and significant change in fluorescence color exhibited by a material upon the application of mechanical force.

#### Tanshinones

Tanshinones are a type of diterpenoid quinone derived from the medicinal herb *Salvia miltiorrhiza Bunge*, commonly known as Danshen in Chinese. While present in various areas of the plant, they are primarily concentrated in the roots [215, 216]. More than 90 tanshinones have been isolated and studied since their discovery in the 1930s [217–220], comprising more than 40 lipophilic and 50 hydrophilic compounds (e.g., protocatechuic and salvianolic acid) [221, 222]. Most tanshinones are red or orange crystals, with their levels closely associated with the color intensity of the roots.

Tanshinones exhibit significant anti-oxidative, anti-inflammatory, antibacterial, anti-tumor, and anti-cardiovascular, and cerebrovascular disease benefits, among others [223–229]. Tanshinone IIA (TIIA) (Fig. 2e), one such tanshinone, modulates apoptosis activation and cell proliferation via the upregulation of stress-mediated proapoptotic proteins calreticulin and caspase-12 [230], while also inhibiting platelet aggregation and activation by regulating G protein and related signaling molecules [231]. Poor water solubility, however, undermines the clinical utility of tanshinones, as bioavailability is limited. Accordingly, augmenting hydrophilicity remains a priority. The introduction of sodium sulfonate base significantly improves not only the hydrophilicity but also the suitability of TIIA for treating cardiovascular diseases [232–235].

#### Hypericin

Hypericin (C_30_H_16_O_8_) is a dianthraquinone (Fig. 2e) that occurs naturally in *Hypericum perforatum Linn*, and is accompanied by derivatives such as pseudohypericin, protohypericin, and hypericodehydrodianthrone [236–238]. The compound forms a large conjugated system through benzene rings and double bonds outside the ring, manifesting as yellow to light brown powder crystals with a peculiar odor [239, 240]. Hypericin is not soluble in water but readily dissolves in polar solvents like pyridine and organic amines, along with alkaline aqueous solutions. Sensitivity to light and heat causes hypericin solutions to turn red. The compound’s distinctive UV absorption peaks are located at 590 and 550 nm, rendering it a valuable component in several applications [241–243].

Hypericin has been used for centuries in China and Europe to treat trauma and inflammation. As studies and research have progressed, it has been revealed that hypericin exhibits several biological activities, such as antibacterial, antitumor, antiviral, central nervous system inhibition, and sedation [244–248]. The addition of hypericin to health products can boost immunity, making it an important ingredient in German depression treatments [249]. Hypericin also has potential applications in veterinary medicine, with many studies conducted on veterinary products containing hypericin that have shown excellent control effects [250, 251].

The high demand for hypericin is driven by its good biological activity, but its main problems are the lower proportion of natural products and immature synthetic routes (Table 1). The development of simple, mild conditions, and low-cost production routes are the focus of ongoing research on hypericin [252].

**Table 1:** Pharmacological activity and limit of the active monomeric constituents of traditional Chinese medicine

Compound name	Source	Pharmacological activity	Limit	References
Carotenoids	Animals, plant, fungi, algae	Maintain visual function, regulate immunity, and protect skin	Poor water solubility	[24]
Gibberellins	Plant, algae, fungi, and bacteria	Plant hormones that regulate a diverse array of developmental processes	Poor water solubility	[25, 26]
Curcumin	Curcuma longa plants in the Zingiberaceae family	Anti-tumor, reduce inflammation, and antiviral	Poor water solubility and sensitivity to light and alkaline environments	[46–50]
Resveratrol	The roots of *Veratrum puberulum Loes*	Antioxidant, anti-inflammatory, anti-cancer, cardiovascular protection, neuroprotection, anti-osteoporosis, antiviral	Poor solubility in water, low oral bioavailability	[54–69]
Rosmarinic acid	Plants of the Lamiaceae and Boraginaceae families	Antioxidant, anti-inflammatory, antiviral, and antithrombotic effects	Non-definite tropism of target	[76–79]
Anthocyanins	Fruits and vegetables	Anti-tumor, anti-inflammatory, antioxidant, memory-enhancing, blood pressure-reducing, cognition-enhancing, radiation-resistance-promoting, and anti-atherosclerotic effects	Vulnerability to deterioration	[89–92]
Camptothecin	The roots, bark, and fruits of the Camptotheca acuminata Decne	Anti-tumor	Toxicity and limited solubility (not soluble in water)	[117, 118]
Vincristine	Catharanthus roseus	Anti-tumor	Peripheral Neurotoxicity, myelosuppressive toxicity, and P-glycoprotein (Pgp)-mediated resistance	[132, 133]
Berberine	The roots, stems, and leaves of Berberidaceae, Loganiaceae, and Ranunculaceae plant families	Cardioprotective and neuroprotective effects, antibacterial, anti-inflammatory, anti-diabetic, anti-tumor, anti-arrhythmic, reverse tumor multidrug resistanc	Limited solubility (not soluble in cold water)	[138, 143–147]
Evodiamine	Evodiamine rutaecarpa	Analgesia, anti-tumor, antibacterial, and metabolic disease regulation, antithrombotic and vasodilator activity, anti-inflammatory and anti-obesity effects, thermoregulatory and cardiovascular protection	Limited solubility	[160–163]
Piperine	Fruits of Piperaceae plants	Gastric ulcer treatment, hypolipidemic properties, antioxidant activity, antidepressant, analgesic, anti-tumor, bactericidal activity and anticonvulsant	Low water solubility	[176–198]
Emodin	The rhizome of Reynoutria japonica Houtt. and Rheum palmatum L	Detoxify, combat bacterial infections, reduce inflammation, and protect the liver, antidiabetic and anti-tumor	Poor water solubility	[200–210]
Tanshinones	Salvia miltiorrhiza Bunge	Anti-oxidative, anti-inflammatory, antibacterial, anti-tumor, and anti-cardiovascular, and cerebrovascular disease benefits	Poor water solubility	[223–229]
Hypericin	Hypericum perforatum Linn	Treat trauma and inflammation, antibacterial, antitumor, antiviral, central nervous system inhibition, and sedation	Not soluble in wate	[244–248]

## Materials-based nanocarriers in delivery of active ingredients from TCMs

Nanocarriers have emerged as an encouraging strategy for administering therapeutic compounds, including those originating from TCMs, which have been utilized for many years and comprise a variety of biologically active substances with potential therapeutic advantages for several ailments. However, issues concerning their bioavailability and toxicity have impeded their clinical application. By encapsulating TCMs, nanocarriers, such as liposomes, polymeric nanoparticles, and organic nanoparticles, can shield them from decomposition in the biological milieu [253]. In addition, they can increase the solubility and stability of TCMs, resulting in greater absorption and bioavailability.

Nanocarriers have been investigated as a delivery method for different TCMs, such as curcumin and resveratrol. Multiple studies have demonstrated their efficaciousness in targeting specific cells and tissues, prolonging the half-life of TCMs, and minimizing their toxicity. Nanocarriers can also be customized with targeting moieties, such as antibodies or peptides, to enhance their specificity towards diseased tissues or cells. Consequently, greater concentrations of TCMs can be achieved at the site of action, leading to improved therapeutic outcomes. The use of nanocarriers offers several benefits for delivering active ingredients from TCMs, including heightened bioavailability, reduced toxicity, and enhanced therapeutic effectiveness. Therefore, they represent a promising platform for developing novel TCM-based treatments for a range of diseases.

### Lipid nanoparticles

**Fig. 3:**
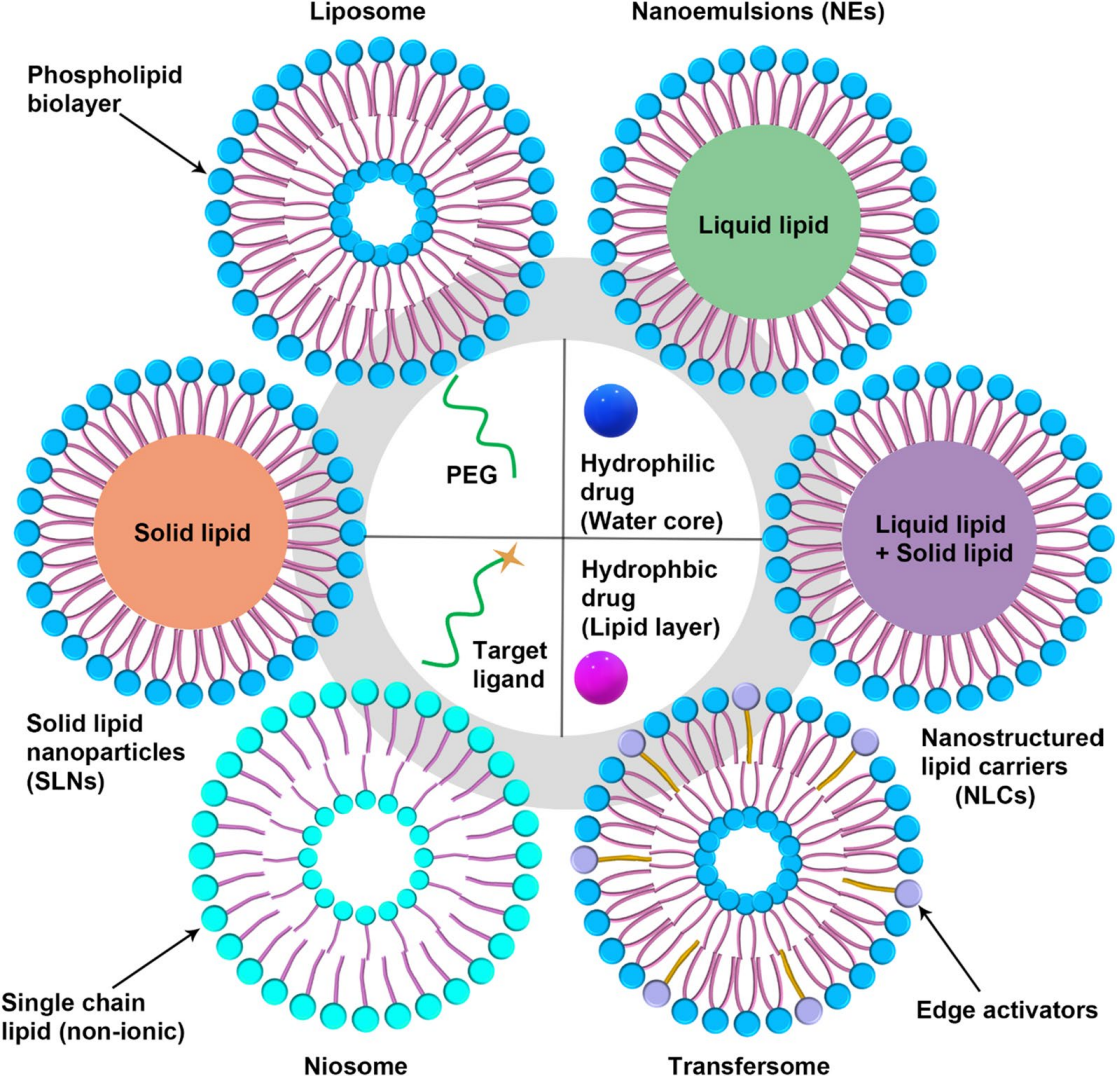
Schematic illustration the formation and application of LNPs

Lipid nanoparticles (LNPs) have emerged across the pharmaceutical industry as promising vehicles to deliver a variety of therapeutics [254]. Lipid nanocarriers can be categorized into various types depending on their method of preparation and physicochemical characteristics, including liposomes, niosomes, solid lipid nanoparticles (SLNs), nanostructured lipid carriers (NLCs), nanoemulsions (NEs), transfersome and ethosome [255], as shown in Fig. 3. LNPs have been expressed and designed to vary in their composition and applications (for example, long-circulating, pH-sensitive, light-sensitive, temperature-sensitive, magnetic-response and enzyme-sensitive) [256], and on the basis of preparations (such as extrusion techniques, reverse phase evaporation method, sonication, and dehydration method). The size of LNPs differs between 0.025 and 2.5 µm, from very small to large vesicles, respectively. Furthermore, LNPs which may possess one or bilayer membranes are classified into unilamellar vesicles (ULV), multilamellar vesicles (MLV), and multivesicular vesicles (MVV) [257].

LNPs are regarded as the ideal nanocarriers based on various advantages [258], including: (1) excellent compatibility with biofilms; (2) easy to modify other groups to enhance targeting and improve drug efficacy, and reduce toxic side effects, such as PEG; (3) hydrophilic and hydrophobic drugs can be encapsulated in different regions.

#### Liposomes

Lipids constitute essential constituents in nanocarrier formulations and play a critical role in the in vitro and in vivo performance of nanocarriers by virtue of their structural similarity to cell membranes having vesicular structures with lipid bilayers [259–261]. The inclusion of active compounds from TCMs can influence tissue distribution, enable liposomes to target designated sites, augment therapeutic efficacy, and diminish toxicity [262]. Liposomes have many advantages, including high biocompatibility, low cytotoxicity, and simple manufacturing processes, chemical versatility for hydrophilic, amphiphilic, and lipophilic compounds, and facile modulation of pharmacokinetic properties by compositional mediation [263–265].

Liposomes are considered an ideal nanocarrier due to their beneficial properties, such as enhancing the solubility and stability of hydrophobic components of TCMs, which are fundamental challenges for the broad application of TCMs [266]. Pathak et al. demonstrated that curcumin can be effectively nano-encapsulated in self-assembled nanocarriers of biodegradable lipids and multimers, which can overcome these obstacles [267]. Elmowafy and colleagues developed and described high-phospholipid lipid nanocarriers loaded with genistein [268]. The aim was to augment the oral bioavailability and pharmacodynamic performance of the compound. The influence of phospholipid contents (1% to 10%) on physico-chemical properties, drug release, and stability were studied using the emulsification/sonication technique.

Temporal persistence is as important for nanocarriers as spatial fidelity since clinical application is severely limited due to insufficient aggregation and short retention time of TCMs drugs in vivo. Pan et al. reported that negatively charged nanocarriers containing retinol had a higher rate of absorption and a longer residence time in the liver, while the silybin content in nanoparticles significantly reduced deposition in the lungs and increased uptake by the liver [269]. Notably, lipid nanocarriers achieved 2 to 3 times greater distribution of silybin in the liver than the free control, and retinol-loaded lipid nanocarriers also exhibited improved liver-specific selectivity. Consequently, it is crucial to optimize lipid nanoparticle formulations to achieve maximal liver targeting.

Liposomes can modify drug pharmacokinetics and biodistribution, mitigate toxicity, and enhance the therapeutic index. A self-assembled blend comprising of the amphiphilic lipid glycerol monooleate (GMO) and the amphiphilic polysorbate 80 (P80) was formulated [270]. The prepared BJO-2 system achieves the optimal drug formulation in nanocarriers, and BJO-cavernosum significantly increases the number of apoptotic cancer cells, due to the enhanced bioavailability of drugs encapsulated in cavernosum. The proposed nanotechnology shows great potential for transformation in TCMs.

The potential of blueberry anthocyanins (ACNs) nanoliposomes coated with pectin were explored as a delivery system. The researchers reported that ACNs were slowly released (≤ 35.9%) in simulated gastric juice but had a more rapid release in simulated intestinal fluid. This was because of trypsin degradation of the vesicles, providing evidence of controlled release characteristics [271]. Similarly, liposomal micelles were discovered to be effective in improving the stability and resistance of blueberry anthocyanins throughout the gastrointestinal tract, leading to a bioavailability of over 90% [272]. To further refine the stability and antioxidant activity of ACNs, nanocomplexes were formulated using intermolecular interaction between chondroitin sulfate and anthocyanin binding at a ratio of 10/1. The resultant structure exhibited stability with an average size of 300 nm, a loading capacity of 6.3%, and a loading efficiency of 99%. The nanocomplexes effectively protected ACNs from degradation at pH 9 and preserved their antioxidant activity, highlighting their potential as a delivery system for ACNs [273]. ANCs persist as an alluring target for the creation of nutraceuticals and functional foods with plausible health benefits, implying the necessity for further investigation in this field.

Liposomes possess the ability to co-deliver numerous drugs of varying polarity and encourage the synergistic impact of these drugs [274, 275]. For example, the alliance of TIIA and glycyrrhetinic acid (GA) showcased more effectiveness than individual components in liver fibrosis, resulting in a desirable encapsulation efficiency of over 80% [276]. Triple-loaded liposomes resulted in significant inhibition of human hepatic stellate cells (HSCs). Ochi et al. simultaneously encapsulated GA and silybin into nanoliposomes by a thin-film hydration method, in which the bioactivity of free drug and stability of silybin were greatly enhanced by the co-delivery of liposomes, resulting in a significant synergistic effect on HepG2 cells [277].

As discussed above, liposomes demonstrated excellent performance as the delivery of active compounds from TCMs. However, the stability of liposomes could be influenced by the in vivo environments (such as pH, temperature) and their own characters (such as size, surface charge, lipid composition). These parameters also impact their loading capacity, the circulation time in blood and targeting efficiency. In order to improve the performance of liposomes in clinic applications, further effort should be focused in the development of assemble technology and efficient selective targeting.

#### Solid lipid nanoparticles

Solid lipid nanoparticles (SLNs) are a category of nanoparticles comprising of a solid lipid core encircled by a surfactant layer, usually under 500 nm in diameter, and are considered a form of nanotechnology [278, 279]. Due to their potential applications in drug delivery, cosmetic products, and the food industry, SLNs have garnered significant attention [280–282]. They provide benefits like ameliorated drug stability, escalated bioavailability and tissue targeting, controlled release of medications, and ameliorated pharmacokinetic profiles. The solid lipid core of SLNs is often composed of biodegradable and biocompatible lipids such as triglycerides, diglycerides, or fatty acids [283]. The surfactant layer is typically made up of non-ionic surfactants such as poloxamers, polysorbates, or phospholipids, which stabilizes the nanoparticle and prevents aggregation [284].

Solid lipid core provides an opportunity to incorporate drug molecules, enabling directed drug liberation and prevention of degradation. Further, SLNs can be surface-tailored with targeting ligands like antibodies, peptides, or aptamers to develop high cell/tissue specificity. In comparison to other nanoparticle systems, SLNs present several benefits, including easy production via conventional techniques (such as high-pressure homogenization, microemulsion, or solvent extraction), low toxicity, and biodegradability. As such, SLNs are a promising option for biomedical applications [285].

Although SLNs have gained attention as a drug delivery system, they do have some limitations such as poor drug loading capacity, drug leakage, and instability during long-term storage, especially with hydrophilic drugs. To overcome these challenges, SLNs can be combined with other nanoparticle platforms like polymeric nanoparticles and liposomes. For example, an oral delivery system with a redox-sensitive CPT prodrug loaded with SLNs could demonstrate potent anticancer activity and increased Caco-2 cell absorption [286]. Nonetheless, the oral bioavailability and intestinal safety of CPT-SS-PA SLNs were first evaluated by in vivo pharmacokinetic and histopathological studies, respectively.

In view of these drawbacks, ongoing studies should be aimed at improving SLN’s performance and nullifying their restrictions, which make SLNs continue to show promise in delivery for TMCs and other drugs with potential applications in various fields.

#### Nanostructured lipid carrier

While SLNs have numerous advantages, they also have some disadvantages, such as low ability to load hydrophilic drugs and the potential for defects in drug ejection during storage. Nanostructured lipid carriers (NLCs) were developed as second-generation lipid nanoparticles to overcome the limitations of SLNs [287, 288]. The structure of NLCs incorporates a blend of solid and liquid lipids in controlled concentrations, resulting in better retention of biological activity when compared to SLNs [289]. NLCs provide larger loading areas, greater capacity for drug loading, and better drug retention during storage. Furthermore, NLCs provide augmented loading capacity and larger loading areas, facilitating better drug retention during storage.

Incorporating multiple active ingredients from various herbs into NLCs can significantly enhance the therapeutic efficacy of drugs such as silasco, glyburic acid, triptolide, resveratrol, among others [290–292]. In a recent study, co-delivery systems for vincristine and temozolomide were developed using two distinct lipid nanocarriers, demonstrating superior tumor inhibition and highlighting the potential of NLCs as potent vehicles for synergistic drug chemotherapy in vivo [293]. Elgizaw et al. developed NLCs loaded with resveratrol and chitosan-coated nano-lipid carriers (CSNLCs) to evaluate their anticancer properties. Their research findings suggested that CSNLCs exhibit significant antitumor and apoptosis-inducing effects, highlighting their potential as a novel drug delivery approach for cancer treatment [294].

Artesunate (ART), a derivative of the TCM artemisinin, has shown promise in cancer treatment due to its potent antitumor activity. However, the low solubility and short half-life of ART limit its clinical application. Tran et al. developed a novel sodium ART lipid carrier (ART-NLCS) and studied its efficacy against human breast cancer cells MCF-7 and MDA-MB-231 both in vitro and in vivo [295]. Their evaluation found ART-NLCS to significantly enhance the anti-cancer activity of free ART by inducing a significant increase in the apoptosis rate of MCF-7 and MDA-MB-231 cells. Similarly, Wang et al. reported on the superior anticancer activity of a novel curcumin-loaded nanostructured lipid carrier (Cur-NLC) in inhibiting proliferation and inducing apoptosis of human liver cancer HepG2 cells compared to natural curcumin. Cur-NLC was found to activate the exogenous apoptotic pathway through regulation of DR5/caspase-8/-3-mediated HepG2 cell apoptotic pathway [296]. Lastly, Rahman et al. studied the potential of nano-lipid carriers of ganoderic acid in cancer prevention and treatment. Their molecular docking and pathway analysis found that ganoderic acid interfered effectively with various signaling proteins involved in cancer pathogenesis, thereby preventing progression of the disease. The study also found that ganoderic acid can regulate liver and non-liver parameters through multiple mechanisms, conferring a chemoprotective effect against diethylnitrosamine-induced liver cancer. These findings suggest that ganoderic acid, when delivered through nano-lipid carriers, may represent a promising therapeutic option for cancer treatment and prevention [297].

Various experiments have demonstrated that the utilization of NLCs methods enhances the stability of both the drug and the carrier, surpassing other lipid systems [298–301]. The similarity of the lipid structure of the NLCs carrier to our biofilms makes it a preferred choice over polymer systems for drug delivery. NLCs’ multifunctional drug delivery system enables facile drug administration to resistant tumors and the central nervous system; hence it’s applied for various cosmetics and chemotherapeutic agents’ delivery [302, 303].

Thus, it can be seen that NLCs overcome the limitations of SLNs and offer more benefits, including their biocompatibility, enhanced biological applicability, stability, and higher drug loading capacity, demonstrating greater potential for application as drug delivery.

#### Microemulsion and nanoemulsion

Microemulsions (MEs) and nanoemulsions (NEs) are rapidly emerging as versatile platforms for drug delivery and biomedical applications [304, 305]. These unique systems are well-suited for a range of applications, from improving drug solubility to enhancing therapeutic efficacy [306]. MEs are isotropic emulsions that are thermodynamically stable and self-assembling, characterized by nanometer size, low viscosity, and self-assembled structures. The emulsions consist of an oil phase, a surfactant, and an aqueous phase as primary components, and possess surface-active molecules that self-assemble. Contrarily, NEs are kinetically stable yet thermodynamically unstable systems with small droplet sizes, excellent resistance to droplet aggregation, creaming, and gravity phase separation [307].

Encapsulation of active drugs within MEs or NEs can result in the development of nanocarriers. These nanocarriers provide several benefits due to the greater specific surface area that facilitates the transfer of encapsulated molecules across cell membranes [308, 309]. As an illustration, to improve bioavailability through the rapid and efficient cellular uptake, Simion et al. incorporated curcumin into peptides containing NEs [310].

#### Ethosomes and transfersomes

Transfersomes are supersonic elastic bilayer vesicles composed of phospholipids and edge activators, such as surfactants [311–313]. These adaptive vesicles are capable of extruding into pores by self-deformation and reformation after passing through pores, thereby crossing skin pores that are smaller than their size [314–316]. Transfersomes are formulated using biodegradable, biocompatible, and non-toxic phospholipids, thereby continuously delivering the active components of TCMs [317]. They serve as carriers for various types of drugs, such as hydrophilic, hydrophobic, high-molecular-weight, and low-molecular-weight drugs. Transfersomes offer controlled delivery of drugs to both the systemic circulation and skin, depending on drug concentration [318].

Ethosomes are a second-generation vesicle system designed to enhance drug delivery [319–321]. These systems contain significant amounts of ethanol (20–45%) and highly elastic phospholipids. Ethanol acts as an osmotic enhancer, and the presence of ethanol negatively charges the vesicles and reduces their size. Interaction with the polar head of lipid molecules elevates lipid fluidity and membrane permeation capacity, inducing a decrease in the melting point of lipids located in the stratum corneum [322]. Consequently, lipids in the skin and elastic vesicles become liquefied [323].

Ethosomes, which are vesicles containing ethanol, have demonstrated outstanding abilities as drug delivery nanocarriers. A recent study was conducted involving the development and characterization of a novel phospholipid nanovesicle, co-hybridized with hyaluronic acid (HA), ethanol, and encapsulated volatile oil drugs (eugenol and cinnamaldehyde [EUG/CAH]), for transdermal drug delivery [324]. The formulation, known as HA-ES, displayed significantly greater stability and percutaneous drug absorption compared to EUG/CAH-loaded ES. Specifically, the stability of HA-ES was 2.5 times more than EUG/CAH-loaded ES, and the percutaneous drug absorption was 1.8 times higher. HA-ES also exhibited exceptional deformability and improved efficacy in UC, demonstrating it as a promising transdermal delivery vehicle for volatile oil drugs.

### Polymer carriers

Novel drug delivery systems rely heavily on polymer carrier materials, which play a critical role in promoting pharmaceutical formulation innovation, intelligent manufacturing, and drug development. There are various drug loading modes for polymer carriers, including covalent conjugation to form polymer-drug conjugates, polymer micelle encapsulated drugs, polymer vesicle encapsulated drugs, and drugs dispersed in polymer gels [325–328]. Polymer carriers possess noteworthy characteristics, such as precise biocompatibility, low toxicity and low antigenicity, controllable drug loading capacity and release behavior, targeted drug distribution to specific cells or organelles, improved drug efficacy, reduced side effects, and expanded use for chemical drugs, protein drugs, peptide drugs, and nucleic acid drugs [329]. Commercially available polymer carriers include Risperdal Consta®, Trelstar®, Sandostatin LAR®, Eligard®, Genexol®, Nanoxel® and Somatuline Autogel®, etc. [330].

#### Polymer micelles

**Fig. 4:**
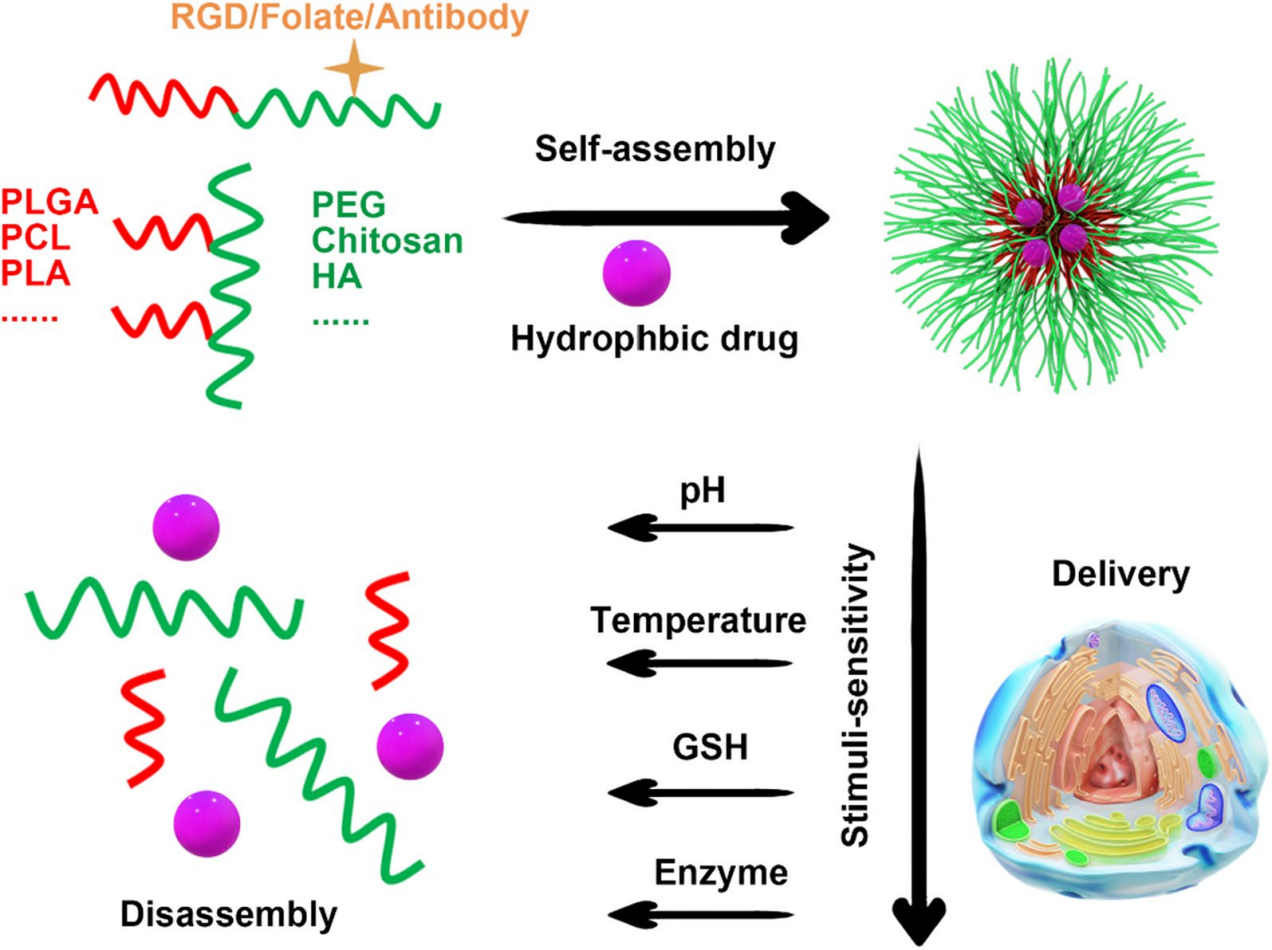
Schematic illustration the formation and application of polymer carriers

Polymeric micelles (PMs) are nanocarriers with core–shell structures, which are formed by the self-assembly of amphiphilic block copolymers in aqueous solutions (Fig. 4) [331]. The hydrophobic core of PMs can encapsulate lipophilic chemotherapeutic agents, such as paclitaxel (PTX), to enhance drug solubility. Meanwhile, the hydrophilic shell, forming a hydration shell, serves as a protective barrier, which minimizes protein adsorption and clearance by the reticuloendothelial system, thus resulting in an extended half-life of drugs. PMs typically exhibit a small particle size range of 10–100 nm with a narrow size distribution, which can be controlled by regulating the length of the hydrophilic blocks. For example, Genexol® PMs, which are clinically approved, utilize methoxy polyethylene glycol-poly (d,l-lactic acid) (mPEG-b-PDLLA) amphiphilic block copolymers to form spherical micelles for PTX encapsulation, offering a promising treatment for ovarian and non-small cell lung cancer [332].

The spherical nanoparticles with regular diameters ranging from 110 to 180 nm were developed by using cross-linked xanthan gum, which were found to be responsive to reducing conditions, resulting in increased drug loading rates and avoidance of drug leakage. In vitro experiments showed that drug release from the nanoparticles could be controlled by pH and reducing conditions, which mimic the internal environment of tumor cells. The nanoparticles were biocompatible, and this study suggests that they have potential as anticancer drug carriers for targeted release [333]. Recently, Huile Gao groups developed some polymer-based nanocarrier to delivery drugs from TCM. For example, a pH-sensitive supramolecular nanosystem with chlorin e6 and triptolide was designed to co-delivery for chemo-photodynamic combination therapy [334]. The combined application of PDT and chemotherapy achieved a great inhibitory effect on tumor growth in H22 and B16 tumor xenograft models with minimal side effects. And they also prepared an Azo reductase-triggered nanocarrier for curcumin delivery in anti-ulcerative colitis treatment [335] and an acidic tumor microenvironment sensitive crosslinked micelle to deliver rosmarinic acid for photodynamic therapy [336].

The unique properties of PMs, such as biocompatibility, inner drugs protecting, target drug delivery, increasing drug circulation time, make them a promising delivery system for TCMs. However, the understanding of their assembly mechanisms and in vivo fate of PMs as drug-delivery is still in the early stage, more efforts need to narrow the gap between lab bench and clinic application.

#### Polymer vesicles

Polymeric vesicles are a type of hollow spheres that self-assemble in solution and resemble liposomes. They are formed from amphiphilic block copolymers [337, 338]. Specifically, the hydrophobic segments of the block copolymers form a membrane layer in the middle of the vesicle, while the hydrophilic segments form brush-like structures on the inside and outside of the membrane [339]. This unique structure creates a hydrophilic inner cavity and hydrophobic membrane, making it an ideal environment for encapsulating both hydrophilic and hydrophobic cargo. By tuning the structure, type, and molecular weight of the block copolymers, vesicles can be engineered to possess specific characteristics and functions to target different diseases. Compared to lipids, block copolymers offer greater synthetic versatility, enabling researchers to design and tailor vesicles for a wide range of applications, including drug delivery, food industry, cosmetics, and medical diagnostics [340–342].

Polymeric vesicles are a sought-after platform for TCMs given their distinctive structure that allows for high-efficiency encapsulation of hydrophilic and hydrophobic drugs. A typical approach for producing drug-loaded vesicles involves directly adding the drug during the vesicle formation process. For example, a novel method was reported as "direct hydration", which involves combining solvent dispersion and homopolymer addition to encapsulate biomacromolecules [343]. Similarly, the loading of hydroxychloroquine (HCQ) and tunicamycin (Tuni) was accomplished by both hydrophilic and hydrophobic anticancer drugs respectively into the lumen and membrane layer of polymer vesicles (poly (ethylene glycol)-b-poly (propylene thioether) (PEG-b-PPS)) [344]. The vesicles loaded with drugs could specifically accumulate within tumor tissue using the EPR effect, and enter cells through endocytosis. The co-delivery of HCQ and Tuni enabled simultaneous induction of ER stress and blockage of autophagic flux, resulting in potent antitumor effects and inhibition of metastasis. Ahmed et al. co-encapsulated two drugs, PTX and DOX in PEG-PLA/PEG-PBD hybrid vesicles [345]. These vesicles were administered to mice with pre-implanted tumors via intravenous injection. The study findings indicated that the highest dose of loaded polymer vesicles tolerated by mice was significantly greater than the combined administration of DOX and PTX monotherapies. Additionally, the vesicles exhibited enhanced efficacy in causing tumor tissue death when compared to monotherapies, which implies their potential as a promising multi-drug delivery approach.

Polymeric vesicles are a sought-after platform for TCMs given their distinctive structure that allows for high-efficiency encapsulation of hydrophilic and hydrophobic drugs. Despite better self-assembly technique, more novel and integral functionalization, and simpler preparation protocols have been explored, investigation on the mechanism and massive production of homogeneous polymeric vesicles could promote translating them into TCMs delivery.

#### Polymer hydrogels

Polymer hydrogel-based drug delivery systems have emerged as a promising method for targeted and prolonged delivery of TCMs because of their exceptional biocompatibility, adjustable physicochemical properties, and capacity to encapsulate both hydrophilic and hydrophobic drugs [346–349]. Designed as three-dimensional network structures, hydrogels can absorb and retain substantial quantities of water or biological fluids while maintaining their structural integrity. These attributes make hydrogels fitting candidates for sustained-release drug delivery systems.

Numerous investigations have explored the possibility of utilizing polymer hydrogel-based systems for TCMs delivery. As an example, a chitosan-based hydrogel with *Panax notoginseng* saponins displayed extended drug release and improved skin penetration [350]. Comparably, a temperature-responsive hydrogel was formulated by conjugating poly(*N*-isopropylacrylamide) and PEG, which could serve for the transdermal delivery of an artemisinin derivative [351]. The study outcomes indicated that the hydrogel could retain the drug concentration at a therapeutic level for as long as 10 days.

In addition to transdermal delivery, polymer hydrogel-based systems have been investigated for the oral and nasal delivery of TCMs. For instance, a mucoadhesive hydrogel-based system was designed for delivering BBR hydrochloride via the oral route, which exhibited prolonged drug release and boosted intestinal permeability [352]. And the nasal delivery of a Chinese herbal formula was explored using a thermo-sensitive hydrogel composed of poloxamer and chitosan [353]. The hydrogel promoted extended release and increased nasal absorption of the active constituents.

Polymer hydrogel-based drug delivery systems present a hopeful approach for the focused, prolonged, and directed delivery of TCMs, owing to their abilities to improve drug bioavailability, diminish adverse effects, and augment therapeutic effectiveness. Ongoing investigations are expected to enhance the clinical usage of polymer hydrogel-based drug delivery systems, and make a significant contribution to the development of new and inventive TCMs formulations.

### Inorganic nanocarriers

Inorganic nanocarriers have surfaced as a promising platform for drug delivery because of their distinct physicochemical characteristics, including tunable size, high surface area, and surface charge [354–356]. These features allow the effective encapsulation and controlled release of a wide array of therapeutics, including nucleic acids, proteins, and small molecules. Gold nanoparticles (Au NPs) and mesoporous silica nanoparticles (MSNs) are common inorganic nanocarriers employed to deliver TCMs [357, 358]. Au NPs exhibit great potential for localized drug delivery and imaging owing to their exceptional optical properties. In contrast, MSNs are characterized by their high drug-loading capacity and adjustable pore size, which enable effective drug encapsulation and release [359, 360]. Inorganic nanocarriers may be functionalized with various targeting moieties, such as peptides, aptamers, and antibodies, to boost their specificity to diseased tissues or cells. Additionally, stimuli-responsive groups, such as pH or temperature-sensitive polymers, may be grafted onto inorganic nanocarriers to achieve controllable drug release in response to specific stimuli. Inorganic nanocarriers may also be functionalized with imaging agents, such as magnetic resonance contrast agents or fluorescent dyes, to enable real-time monitoring of therapeutic response and drug delivery.

#### Au NPs

Au NPs exhibit distinctive benefits over bulk gold because of their biocompatibility, stability, unique interaction with light, small size, and affinity towards biomolecules [361]. Localized surface plasmon resonance (LSPR) alludes to the absorption and scattering of light by Au NPs when exposed to a particular wavelength. Scientists have consequently designed a variety of drug delivery systems based on these advantages of Au NPs.

A novel Au NPs (Do-Au NPs) was successfully synthesized from the aqueous extract of Dendrobium officinale (DO), a TCM, with good antitumor efficiency, without the increasement of in vivo and in vitro toxicity [362]. This investigation offers crucial knowledge for the creation of innovative nanomedicines for liver cancer therapy. Apart from herbal extracts, researchers have investigated other techniques for enhancing the solubility and bioavailability of promising clinical candidates, such as licochalcone A [363]. Sun and colleagues have successfully loaded licochalcone A onto hollow Au NPs (L-HGNPs) using ultrasound, resulting in a significant improvement in their solubility and bioavailability [364]. Bao et al. successfully synthesized AuNPs using *Pholiota adiposa* polysaccharide (PAP-1a) without the requirement of additional chemical agents [365]. The study results reflect that PAP-AuNPs exhibit markedly enhanced capabilities in immune modulation and anti-tumor efficacy when compared to PAP-1a administered alone. The PAP-AuNPs have also demonstrated excellent biocompatibility both in vitro and in vivo with no toxic implications. AuNPs were synthesized by Ling and colleagues using the root extract of *Paeonia montana* (PM-AuNPs), a woody plant that is extensively utilized in TCMs for treating diverse ailments. The study findings revealed that PM-AuNPs exhibit desirable properties required for an effective nanodrug candidate, and possess significant potential for curtailing inflammation in murine microglial BV2 cells in vitro. Furthermore, the conducted in vivo experiments validated that PM-AuNPs could mitigate neuroinflammation and improve motor coordination in mice with Parkinson’s disease [366].

Au NPs represent a promising platform for the targeted delivery of TCMs owing to their unique chemical and physical properties, including well-controlled size, biocompatibility, high surface area-to-volume ratio, and customizable surface functionalization. However, the use of AuNPs in TCMs delivery poses several challenges and considerations that need to be addressed [367, 368]. One primary challenge pertains to the quality control and standardization of TCMs formulations. TCM involves complex mixtures of compounds with varying chemical properties and biological activities. The lack of quality control and standardization in TCMs preparations might affect the Au NP synthesis or have unpredictable effects on the pharmacological properties of the final product, necessitating a strict quality assurance program. Another significant consideration is the potential toxicity of Au NPs and interactions with TCMs components. Although Au NPs possess good biocompatibility, larger sizes and non-optimal surface modifications can lead to adverse effects in vivo. Moreover, the interactions between TCMs ingredients and Au NPs might alter their pharmacokinetics or potentiate unintended side effects [369]. Additionally, the variability in the clinical efficacy of TCMs due to individual differences in genetic backgrounds, lifestyles, and therapeutic interventions can pose additional challenges to Au NPs-mediated TCMs delivery. Thus, carefully designed preclinical studies to evaluate the safety and efficacy of Au NPs in TCM delivery systems are essential to ensure that they meet regulatory and clinical requirements.

It can be seen that the use of Au NPs-mediated TCMs delivery represents a promising strategy, but the variability in the clinical efficacy of TCMs brings additional challenges, such as individual differences in genetic backgrounds, lifestyles, and therapeutic interventions. Thus, carefully designed preclinical studies are essential to evaluate the quality control, toxicity, and therapeutic efficacy of Au NPs in TCM delivery systems, ensuring that they can meet regulatory and clinical requirements. Meeting these challenges would significantly accelerate the development of new TCM formulations and pave the way for the targeted delivery of TCM using Au NPs.

#### MSNs

Due to their unique hollow structure, easy surface modification, good biocompatibility and large specific surface area, MSNs have become highly sought-after drug carriers in cancer research [370, 371]. The encapsulation of resveratrol in MSNs greatly improved its physical and chemical properties, ultimately leading to enhanced biological activity and promising implications for cancer therapy [372]. An MSNs-based nanoplatform was proposed, which loaded with isoalanine (ISOIM) and camouflaged by cancer cell membranes (CCM) as CCMMSNs-ISOIM [373]. The suggested nanoplatform provides numerous benefits, such as immune evasion, anti-phagocytosis, and active targeting at the tumor site, which leads to increased drug delivery and anti-cancer efficacy. Wu and colleagues described a temperature and pH-responsive drug carrier comprising MSNs for delivering two therapeutic agents, EVO, and BBR [374]. The study findings demonstrate that the biocompatible nanocarrier enhances drug efficacy and compatibility while also maintaining optimal drug profiles within the acidic and elevated temperatures characteristic of tumors. Combining EVO and BBR in MSNs present a remarkable synergistic therapeutic outcome, as supported by in vitro and in vivo experiments. These findings represent a positive advancement in the development of efficient drug delivery systems for cancer treatment.

Furthermore, two types of MSNs were designed and they were responsive to redox by utilizing disulfide bonds to attach polyethyleneimine-folic acid (PEI-FA) or HA to the MSN surface [375]. These biocompatible nanoparticles were efficiently taken up by MDA-MB-231 breast cancer cells and effectively transported curcumin (CUR) to the tumor sites both in vitro and in vivo. The FA-modified MSN exhibited slightly superior targeting capacity than the HA-modified MSN, possibly due to FA’s lower molecular weight, which enables it to easily bind to the receptor. Nonetheless, further investigation is necessary to validate this explanation. The research indicates that the multifaceted MSN vehicles developed here constitute an appropriate and highly efficient drug carrier for breast cancer therapy. Zhang et al. suggested employing MSNs conjugated with FA (FA-MSNs) as carriers for delivering RJ-III, which could decrease the drug’s acute toxicity and enhance its biomedical application by extending drug release and targeted delivery. This study represents the first application of FA-MSNs in the delivery of RJ-III to mitigate its toxicity. The findings suggest the plausibility of delivering RJ-III to inflammatory cells in a targeted manner, thereby enhancing the drug’s efficacy. Additionally, this study provides valuable data for future research to assess the anti-inflammatory activity of RJ-III [376].

Despite notable strides have been made in the development of inorganic nanocarriers for drug delivery, several challenges remain to be surmounted. One of the primary hurdles is ensuring the biocompatibility and stability of inorganic nanoparticles in vivo. Thus, further optimization of surface chemistry and functionalization strategies are imperative to achieve superior biocompatibility and lower toxicity. Additionally, for the sake of optimal performance for clinical applications, further research is necessary to elucidate the mechanism of drug release from inorganic nanocarriers. Owing to the continuous advancements in inorganic nanocarrier technology, these challenges it is expected to be overcome in the near future, leading to the booming of therapeutic approaches inorganic nanocarriers for treating a diverse range of diseases.

### Organic/inorganic nanohybrids

While a variety of nanomaterial delivery systems have been employed to circumvent the barriers to TCMs, single nanomaterials inevitably possess certain limitations. Therefore, constructing different nano-level heterozygotes via reasonable design and effective methods or strategies is expected to overcome the drawbacks of current TCMs carriers and broaden their applications in the biomedical field.

Organic/inorganic nanohybrids pertain to nano-level heterozygotes with unique functions that are synthesized by combining organic materials with inorganic nanoparticles via specific methods [377–379]. In the process of nanohybrids, interaction forces between the two phases, such as electrostatic interaction and hydrogen bonds, will arise. Therefore, organic/inorganic nanohybrids exhibit the advantages of both organic materials and inorganic nanoparticles, such as excellent stability, relative safety, and drug release that responds intelligently to the environment. Additionally, the morphology, size, and function of nanohybrids can also be selectively regulated by adjusting the organic/inorganic allocation ratio, thus overcoming many of the limitations faced by single components during the application process.

**Fig. 5:**
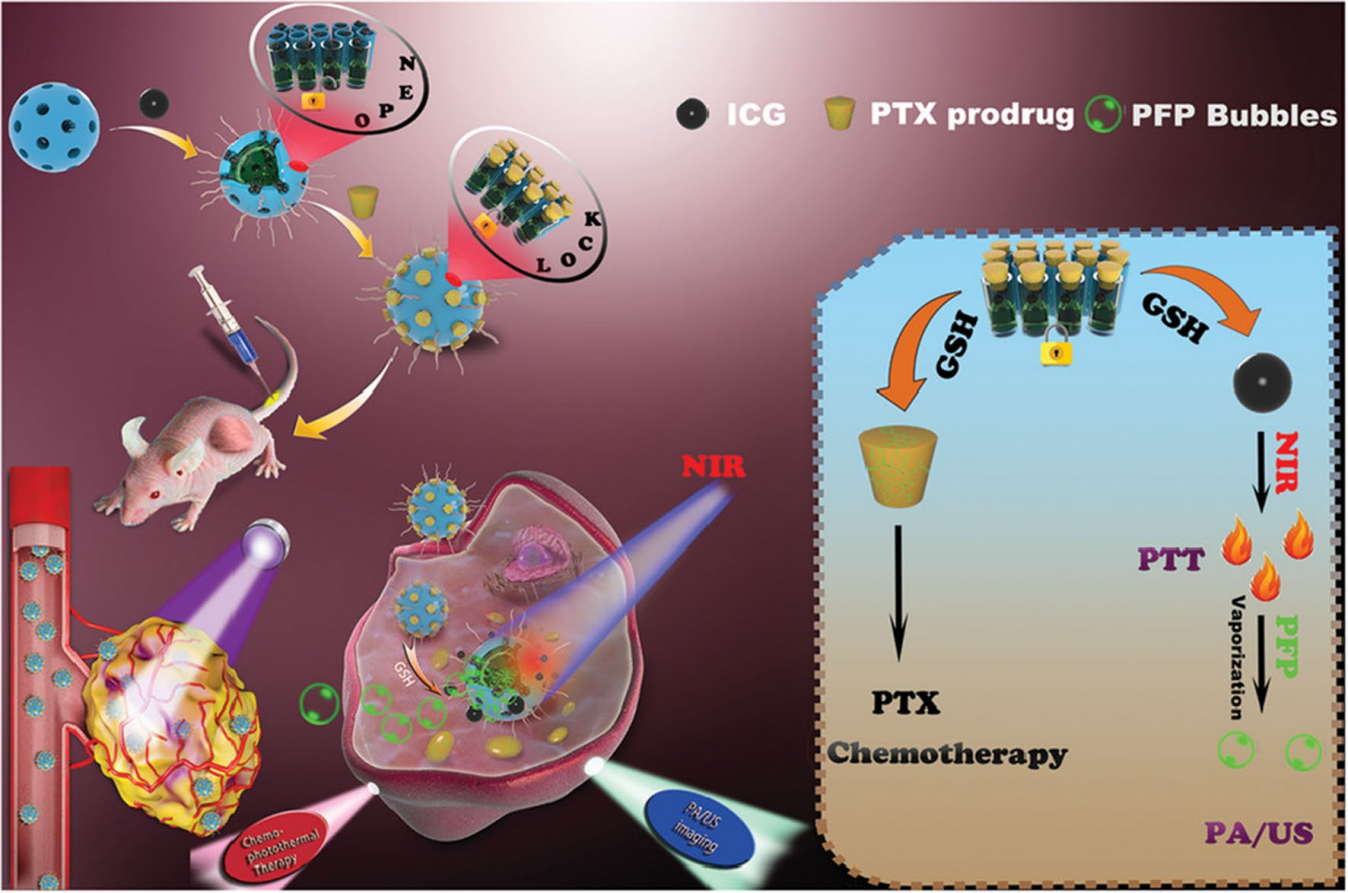
Schematic illustration of the construction of ICG/PFP@HMOP-PEG for imaging and chemo-photothermal therapy [382]

Nanohybrids, namely MSNRMoS_2_-HSA/Ce6, was fabricated for image-guided photothermal and photodynamic combined therapy [380]. The mesoporous silica (MSNR) is deployed as a template to introduce sulfhydryl groups through the reaction with (3-mercaptopropyl) trimethoxysilane (MPTMS), resulting in the formation of MSNR-SH. After encapsulating MoS_2_ on the surface, human serum albumin (HSA), and photosensitizer dihydroporphyrin (Ce6) are introduced through a chemical reaction. The resulting nanohybrid is then activated by 1-(3-dimethylaminopropyl)-3-ethylcarbodiimide (EDC) and hydroxythiosuccinimide (NHS). Liu et al. have modified inorganic Au NPs on graphene oxide surface in situ to achieve good biocompatibility, targeting, and chemotherapeutic synergy [381]. They have also continued to modify PEG and targeted aptamer DNA-AS1411 on their surface and loaded the anti-tumor drug DOX. The results suggest that the nanohybrid possesses good photothermal conversion ability, stability, and targeting, and can effectively kill tumor cells. Furthermore, in vivo photothermal/chemical synergistic anti-tumor effect of nanohybrids has been demonstrated in tumor-bearing mouse models. Others synthesized hollow mesoporous silicon doped with disulfide bonds and loaded perfluoropentane (PFP) and near-infrared dye ICG simultaneously into mesopores [382] (Fig. 5). The researchers have then grafted PTX prodrugs as sealers outside the pores using disulfide bonds to form nanohybrids (ICG/PFP@HMOP-PEG). The experimental results reveal that the nanohybrid has excellent photothermal conversion performance and photothermal stability, and PFP is vaporized under near-infrared light irradiation to achieve ultrasound imaging-mediated synergistic therapy.

A polymer carrier material utilizing MSNs was reported to targeted towards tumors by modifying PEG and FA simultaneously onto the MSNs carriers [383]. The application of hydrophilic PEG improves the biocompatibility and stability of functional nanocarrier materials, while FA confers active targeting of carrier materials to tumor cells. The latter enhances the therapeutic effect of cancer by improving the endocytosis of carrier materials. Additionally, a stable organic/inorganic hybrid polymer vesicle based on organosilicon was developed. This vesicle was loaded with DOX and CPT and shows anti-tumor performance. The system achieves the purpose of synergistic treatment of cancer with a hydrophobic drug molecule, CPT, and hydrophilic drug molecule, DOX·HCl [384]

Organic materials and inorganic nanoparticles compose the organic/inorganic hybrid delivery systems, exhibiting great potential for cancer therapy due to their modifiability, functionality, and long in vivo circulation time. However, current research in this area is still in its early stages and constantly encounters numerous development challenges. Furthermore, the low clinical conversion rate resulting from the diversified and complicated nano-hybrid system components significantly hinders the development of this nano-drug delivery system. In light of these, it is crucial to develop simpler and more efficient methods for producing nano heterozygotes with excellent performance through reasonable design, and expand the clinical applications of the obtained organic/inorganic hybrid delivery systems for future development.

## Nanocarriers based on active ingredients from TCMs

At present, there is an increasing amount of research dedicated to providing nano-delivery systems for delivering individual active molecules of TCMs. Although the complex physical and chemical properties of their components make it difficult for nano-delivery systems to accommodate co-loading of compound drugs [385], recent studies have demonstrated that self-assembly between active molecules of TCMs can form nanoparticles [386–388]. This self-assembly technology has led to a series of research results on nano-formulations based on self-assembly of active molecules of TCMs [389]. Self-assembled nanoparticles have several advantages, including no carrier and fewer adverse reactions, good drug loading capacity, good pharmacokinetics, and the ability to inhibit multidrug resistance and play a synergistic therapeutic effect. At the same dose, the efficacy of self-assembled nanoparticles is better than that of free drug treatment groups. Additionally, self-assembled nanoparticles can be used to construct an integrated intelligent nano-system for diagnosis and treatment [390, 391]. As an emerging field, current research on natural product self-assemblies primarily focuses on exploring the comparison of biological activities before and after self-assembly of new drug self-assemblies and active molecules of TCMs.

### Material-drug conjugates

**Fig. 6:**
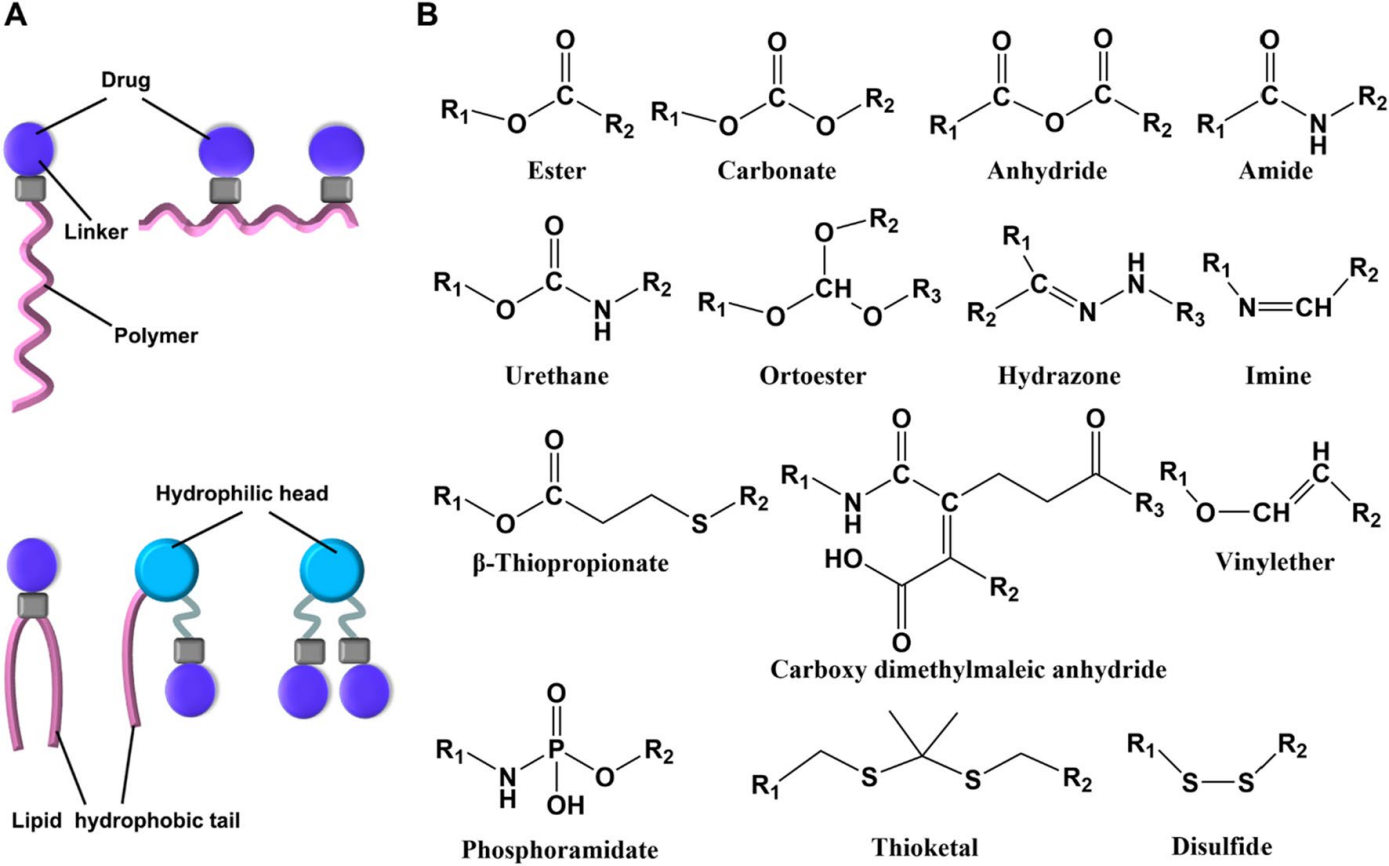
The prodrugs of CTMs and the chemical structures of common sensitive bond used in prodrugs

It is noteworthy that polymer and lipid can hinder drug loading due to intermolecular hydrophobic interactions, leading to drug aggregation, reduced loading capacity, and uncontrollable drug proportions. One approach to enhancing the loading capacity of drugs is to replace donor-receptor interactions between materials and drugs with covalent bonds forming material-drug conjugates. Material-drug conjugates refer to a class of therapeutic agents consisting of a biologically active drug covalently linked to a material molecule [392, 393], mainly including polymer-based prodrug and lipid-based prodrug (Fig. 6A). Material-drug conjugates can be synthesized using either chemical or biologic methods in which the drug and material are conjugated to form a single macromolecule with specific molecular weight and release characteristics. There are some physiological environment-sensitive covalent bonds between materials and drugs, including pH-sensitive, enzyme-sensitive and redox-sensitive covalent bond, as shown in Fig. 6B. Under physiological conditions, sensitive bonds break and free drugs are released. This approach allows for precise control over the drug-material ratio, pharmacokinetics, and pharmacodynamics to enhance drug efficacy and safety [394, 395]. Due to their tunable properties, material-drug conjugates have shown great potential in the treatment of numerous diseases, including cancer, inflammation, and autoimmune disorders.

**Fig. 7:**
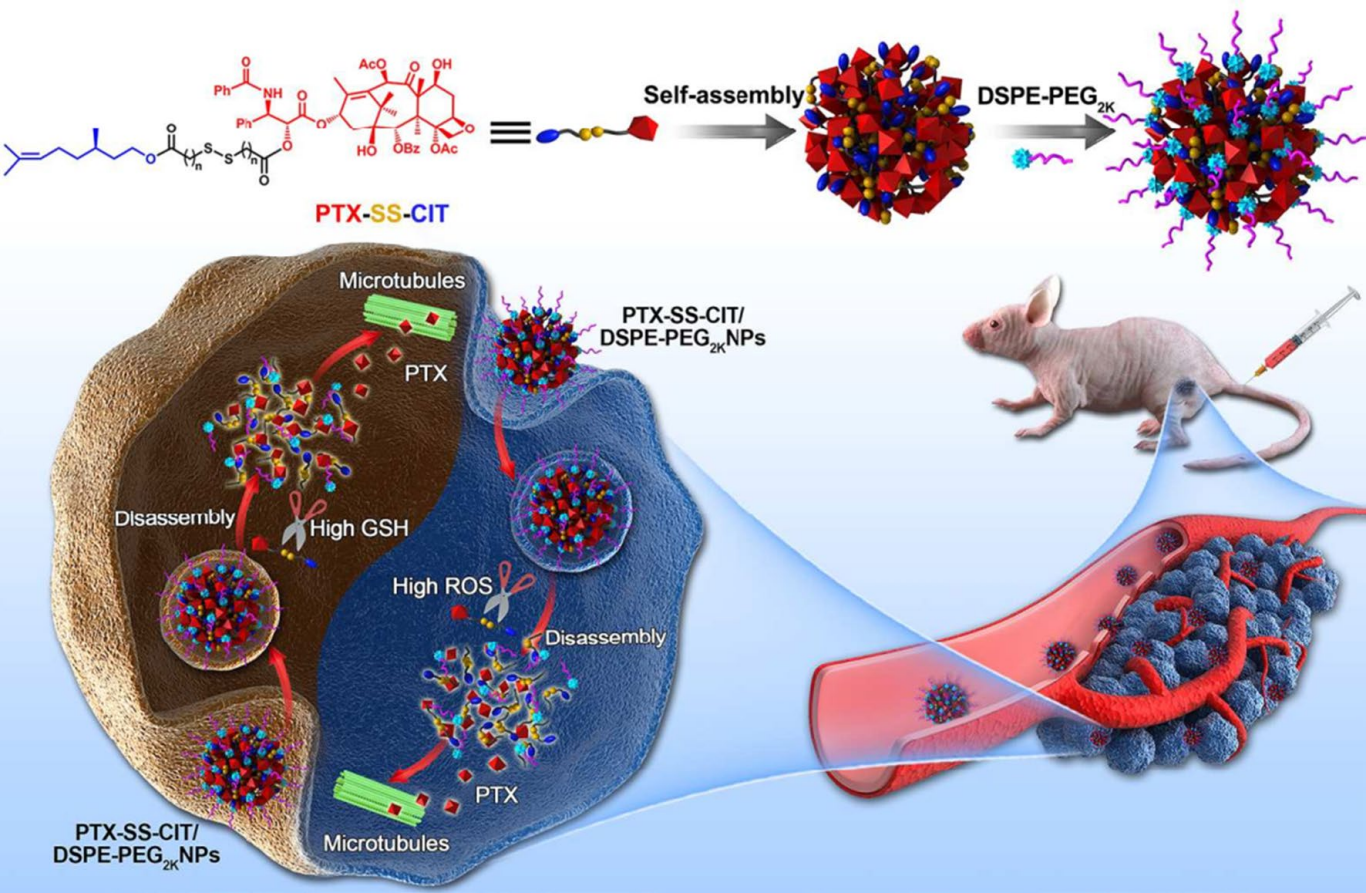
PTX-SS-CIT prodrugs and DSPE-PEG_2k_ were utilized to prepare PEGylated prodrug NPs for tumor therapy [397]

Above all, the prodrug based nanocarriers have many advantages as a potent platform for anticancer drug delivery, such as improved drug availability, high drug loading efficiency, resistance to recrystallization upon encapsulation, and spatially and temporally controllable drug release [396]. The paclitaxel-SS-citronellol conjugates (PTX-SS-CIT) were synthesized by linking via different lengths of disulfide bond containing carbon chain (Fig. 7) [397]. The prodrugs can self-assemble into uniform size nanoparticles with impressive high drug loading (> 55%). Similarly, a novel redox-responsive nanoparticle was developed for targeted delivery of docetaxel (DTX) [398]. The researchers modified a small molecule DTX prodrug with cystamine containing disulfide bonds (Cys-DTX) to create a redox-responsive Cys-DTX/CS-ss-DTX nanoparticle. Use of this nanoparticle system increased DTX release in reducing environments, improved the permeability of tumor tissue, enhanced cytotoxicity and reduced side effects. These findings suggest that Cys-DTX/CS-ss-DTX nanoparticles hold great promise for future cancer chemotherapy.

In a recent study, Fru and colleagues designed polymer-drug conjugates consisting of PEG-betulinic acid (PEG-BTA) and evaluated the cell death, anti-inflammatory, and antioxidant activities of the resulting prodrugs. Their findings indicated that the superior anticancer activity and activation of both the intrinsic and extrinsic pathways of apoptosis were attributed to the conjugated PEG-BTA prodrugs [399]. Similarly, monomethoxy-poly(ethylene glycol)-*b*-poly(lactide) (mPEG-PLA) conjugated DTX through an ester linker and the results shown that the prodrugs exhibited superior anticancer effect than free drug [400]. Furthermore, to improve the target ability, trastuzumab and FA-based multiblock polymer drug conjugates was synthesized via an ester linkage between FA and the hydroxyl groups on the polymer backbone [401]. The prodrugs exhibited good drug loading capacity of 22% and the apoptosis of MCF-7 breast cancer cell was 80% while that of free drug was only 20%.

Apart from the polymer-based prodrug, lipid-drug conjugates also were developed for nanocarriers. Based on the extremely short half-life of Artemisinin and its derivatives (artemisinins) in vivo, two artemisinin derivatives (dAPC and dACC) were synthesized, which possess mimic phospholipids and cationic lipids, respectively [402]. A TNF-α siRNA/artemisinin co-delivery nano-microplex (MTAsi@MG) is further prepared by immobilization of TNF-α siRNA/lipoplex on porous microfluidic HA microspheres, which were in situ injected for Rheumatoid Arthritis Therapy.

### Drug-drug conjugate or drug pair based nanocarriers

“All-in-one” carrier-free-based nano-multi-drug self-delivery system could combine triple advantages of small molecules, nanoscale characteristics, and synergistic combination therapy together. Drug-drug conjugate is a common no-carrier form of prodrug, which often targeting different targets to obtain a combined effect. To release the drug successfully, the covalent bond often is sensitive to pH, enzymes and temperature (Fig. 6).

Methotrexate (MTX) and 10-hydroxyl camptothecin (HCPT) were chosen to conjugate through ester linkage for the combination cancer chemotherapy [403]. Both in vitro and in vivo studies demonstrated that MTX-CPT NPs could specifically co-deliver multi-drug to different sites of action with distinct anticancer mechanisms to kill folate receptor-overexpressing tumor cells in a synergistic way. Furthermore, they developed a dual-targeting delivery system based on 1,2-distearoyl-snglycero-3-phosphoethanolamine-hyaluronic acid (a principal ligand of CD44 receptors)-MTX (a selective ligand of folate receptors) nanoparticles, which was exploited to carry HCPT-MTX conjugate for synergistically boosting dual-drug co-delivery [404]. Similar method also was used to design the conjugate of CPT and chlorambucil through a disulfide linkage, which could be destroyed under the stimulus of glutathione (GSH) [405]. Apart from this, chlorin e6 (Ce6), berberrubine (BBR) and matrix metalloproteinase-2 (MMP-2) response peptide (PLGVRKLVFF) were coupled by linkers to form a linear triblock molecule BBR-PLGVRKLVFF-Ce6 (BPC), which can self-assemble into nanoparticles for chemo-photodynamic therapy of breast cancer [406]. Sometimes, the conjugate could be derived from the same drug. For example, a novel paclitaxel-s–s-paclitaxel conjugate could obtain the high drug loading (∼78%, w/w) by conjugating PTX to PTX using disulfide linkage [407].

In addition to the covalent bond coupling, two drug molecules can form drug pairs through other non-covalent bonds, including van der Waals, hydrogen bond, electrostatic attraction and π-π stacking, etc., which can form nanocarriers by self-assembling manner, and can be used together for disease treatment [389]. The potential of natural phytochemicals in drug delivery, combinational therapeutics, imaging and theranostics may be enhanced by controlling the size, morphology, surface chemistry, internal structures and stability of nanostructures based on natural phytochemicals. For example, to improve the therapeutic effect of PTX limited by its water insolubility and multidrug resistance, Cheng et al. showed that PTX-sulfur-sulfur-BBR (PTX-ss-BBR) NPs can accumulate in mitochondria, owing to the charge and lipid properties of BBR. Molecular dynamics (MD) simulations demonstrated that PTX molecules with multiple phenyl groups were inside the nanostructure, and they were surrounded by BBR. Among them, π-π-stacking and hydrophobic interactions clarified the self-assembly mechanism of PTX-ss-BBR NPs.

Firstly, the self-assembly manner can occur between different molecules of one drug. A pure nanomedicine without a carrier was introduced by incorporating the self-assembly of ursolic acid molecules [408]. The ursolic acid molecules were able to generate stable nanoparticles, varying between 100 and 200 nm in size, through hydrogen bonding and hydrophobic interactions. These nanoparticles displayed a drug loading capacity of up to 60%. The anti-cancer effects of this nano formulation were superior to free ursolic acid, as it effectively hindered cancer cell proliferation and led to apoptosis. The in vivo experiments performed on mice xenografted with A549 cells exhibited significant tumor growth suppression and liver protection using the nano formulation. Another study discovered that pachymaric acid A could self-assemble into an injectable gel with good injection performance [409]. This bioactive gel can be slowly degraded in vivo, not only exerting efficacy through its own pachymaric acid A molecule but also synergizing with other drugs to exert anti-tumor efficacy. Similar manner were also reported, such as honokiol [410], disulfide-modified GSH-responsive BA [411] and ursolic acid [389]. These discoveries suggest that the utilization of self-assembled nanomedicines exhibits a promising avenue for the treatment of cancer.

Secondly, the construction behavior which named as co-assembly also occurs between two different drugs. For example, the stable nanoparticles of baicalin and BBR were successfully prepared to investigate the main active components of this formula [409]. They have illustrated that the self-assembly between BBR and baicalin in an aqueous solution is caused by electrostatic interactions between the quaternary ammonium ion in BBR and the carboxyl group in baicalin. The resulting gel self-assembly increased the antibacterial activity, elongated the half-life of BBR, decreased adverse reactions, and weakened the bitter taste. Similar manner was also used between cinnamic acid and BBR to cure multidrug-resistant staphylococcus aureus [412]. According to other report, self-assemblies made up of naturally occurring compounds derived from plants may serve as a therapeutic strategy to combat bacterial resistance. Among these compounds is cinnamic acid, which is found in cinnamon and possesses numerous medicinal properties such as antioxidant, antibacterial, anticancer, anti-inflammatory, and antidiabetic activities [413]. This acid interacts with free radicals, providing electrons and generating stable products that terminate free radical chain reactions. It does not necessitate the use of any carrier or additives, making it an excellent candidate for drug release with notable biocompatibility. A number of alkyl-modified BBR derivatives were synthesized, which showed significantly better hydrophobicity and antibacterial activity compared to the parent compound [413]. These derivatives could be particularly effective against *H. pylori*, a bacterium that is known to cause a variety of gastrointestinal illnesses.

Importantly, drugs pair often have a combined effect of 1 + 1 > 2, avoiding drawbacks while leveraging each other's strengths. Wang et al. have established a novel methodology to create nano formulations of PTX and ursolic acid using the self-assembly technique. By exploiting the self-assembly characteristics and anti-tumor activity of these compounds, they managed to significantly extend the plasma half-life of PTX and ursolic acid, thereby preventing fast drug leakage within the body. Ursolic acid, with its excellent biocompatibility and specific bioactivity, adds significantly to this approach. The resulting PTX-ursolic acid nanoparticles not only display the anticancer activity of PTX but also uphold the anti-tumor and hepatoprotective effects of ursolic acid, working together in a synergistic manner for therapeutic benefits [414].

**Fig. 8:**
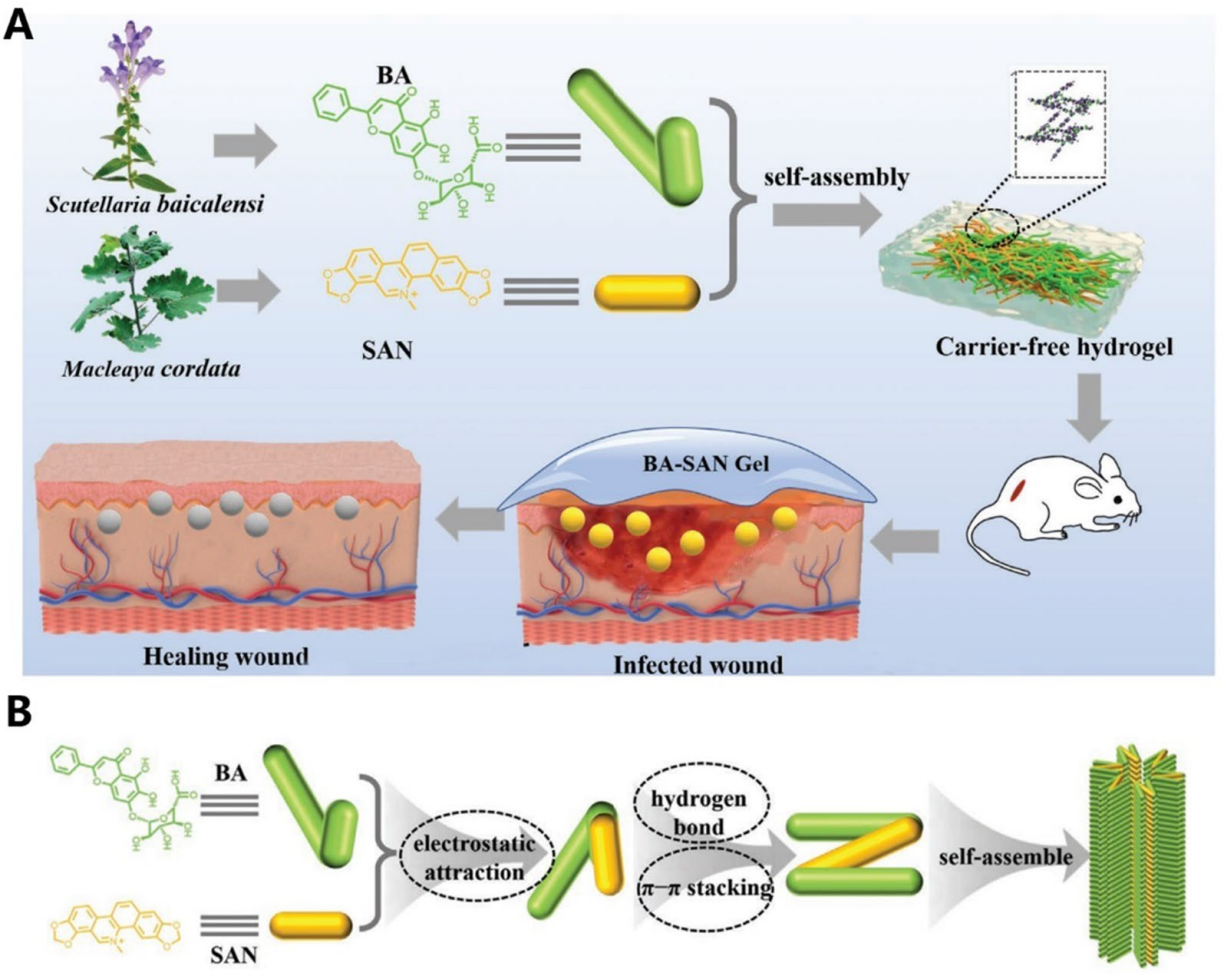
**A** Schematic illustration of the preparation of carrier-free BA-SAN hydrogel and its antibacterial and wound healing effects; **B** Schematic diagram of BA and SAN self-assembly [416]

This groundbreaking method highlights the potential of synergistic drug combinations to improve therapeutic outcomes and reduce any adverse effects, offering a promise of developing innovative, safe, and effective therapeutic agents for cancer treatment. More recently, a study documented the self-assembled supramolecular nanoparticles made using *Radix et Rhizoma Rhei* and *Coptidis Rhizoma*. They found that rhein and coptisine compounds could create nanofibers, holding potential applications in cancer-related studies [415]. Similarly, others reported that the natural star antibacterial and anti-inflammatory phytochemicals baicalin (BA) and sanguinarine (SAN) could directly self-assemble through non-covalent bonds such as electrostatic attraction, π–π stacking, and hydrogen bonding to form carrier-free binary small molecule hydrogel (Fig. 8) [416]. Due to the matched physicochemical properties and synergistic therapeutic effects, BA-SAN gel could inhibit bacterial virulence factors, alleviate wound inflammation, promote wound healing, and had good biocompatibility. The study was helpful to the clinical translation of antibacterial hydrogels due to the potential solvation of unpredictable risks and high costs of carrier excipients.

**Fig. 9:**
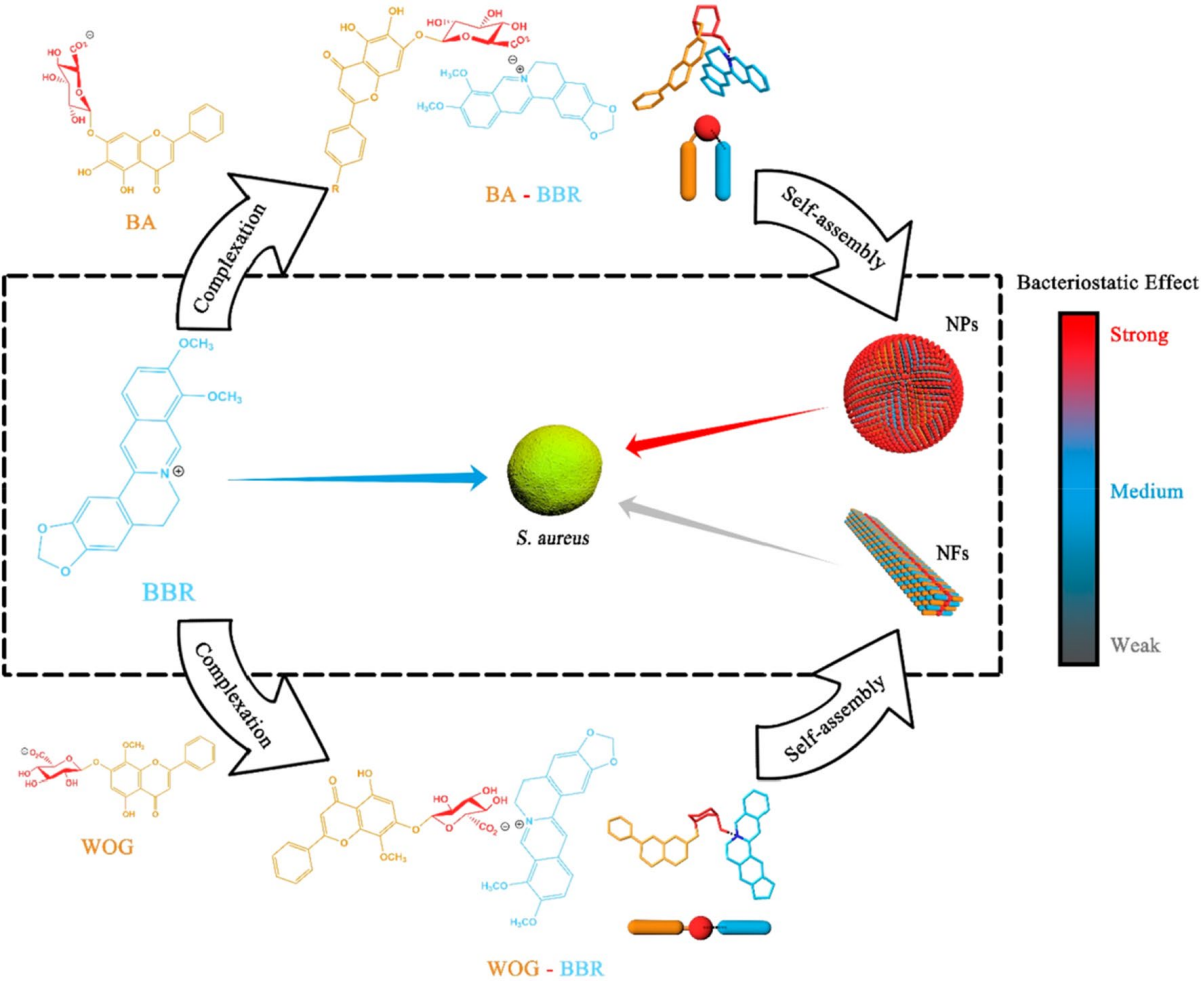
Natural self-assembling mode between BBR and flavonoid glycosides (BA, WOG) and their modified antibacterial application [417]

Interestingly, the conformations of different drug molecules can affect the assembly form of nanostructures, thereby affecting their biological effects. As shown in Fig. 9, two natural self-assembling modes between BBR and flavonoid glycosides: nanoparticles (NPs) and nanofibers (NFs) were reported, which were both mainly governed by electrostatic and hydrophobic interactions [417]. These two nanostructures exhibited different antibacterial properties from BBR. NPs showed significantly enhanced bacteriostatic activity, whereas NFs displayed a much weaker effect than BBR. The distinguishing properties can be attributed to the different spatial configurations and self-assembly processes of NPs and NFs. Flavonoid glycosides and BBR first formed a one-dimensional complex unit and subsequently self-assembled into three-dimensional nanostructures. With the hydrophilic glucuronic acid toward the outside, NPs exhibited stronger affinity to bacteria, thereby inducing the collapse of the bacteria population and the decrease in biofilm. In addition, in vitro hemolysis tests, cytotoxicity tests, and in vivo zebrafish toxicity evaluation showed that the obtained self-assemblies had good biocompatibility. This supramolecular self-assembly strategy can be applied to construct other nanoscale antibacterial drugs and thus provides weapons for the development of self-delivering drugs in bacterial infection treatment.

While nano-formulations based on self-assembly strategies of active molecules of TCMs have shown many advantages, carrier-free nano-formulations based on self-assembly of active molecules of TCMs still have some shortcomings in terms of clinical translation. The effects of TCMs compounds are often multi-drug combinations, which are limited by the self-assembly characteristics of TCMs molecules. At present, nanostructures are restricted to mixing only two drugs, and there are scanty accounts of self-assembly involving three or numerous drugs. The self-assembly force between active molecules of TCMs is mainly based on non-covalent forces, resulting in self-assembly nano-formulations of TCMs molecules often having poor physical and chemical stability. Improving the stability of nanostructures will be one of the keys to promoting their clinical translation. Addressing these challenges will require innovative approaches to overcome the limitations of current self-assembly strategies and expand the potential of TCMs compounds in the development of new therapeutic agents (Table 2).

**Table 2:** Nanocarriers of the main active ingredients in traditional chinese medicines

Nanocarriers	Materials	TCMs	Advantages	References
Liposomes	Lecithin-chitosan lipid polymer	Curcumin	High encapsulation; stable in the pH of 2–6; enhanced antioxidant	[267]
	phospholipid	Genistein	Higher bioavailability; enhanced liver homogenate lipid peroxidation property; histopathological alterations in liver and kidney	[412]
	Hexadecyl palmitate; squalene; soybean phosphatidylcholine;	Silibinin	Slower silibinin release; enhanced therapeutic efficiency and a reduction of adverse responses	[269]
	Lipid glycerol monooleate; polysorbate 80	Brucea javanica oil	Stealth properties and colloidal stability; 90% encapsulation efficiency rate; enhanced anti-tumor efficiency;	[270]
	Phosphatidylcholine; phosphatidylethanolamine; phosphatidylinositol; phosphatidic acid; glycolipids; neutral lipids; chondroitin sulfate	Anthocyanins	Enhanced stability and bioavailability; higher encapsulation efficiency; increased number of apoptotic cancer cells; reduced degradation and preserved antioxidant activity	[271–273]
	Soybean phospholipid; cholesterol	Tanshinone II A	Inhibit the proliferation of human HSCs; sustained release trend	[276]
	mPEG2000-DSPE; Dipalmitoylphosphatidylcholine; lecithin; cholesterol	Glycyrrhizic acid; silybin	Increment of co-entrapment; suitable EE, stability, controlled release of drugs; lower IC50 on the HepG2 cancer cell line	[277]
Solid lipid nanoparticles (SLNs)	Glycerin monostearate; poloxamer; CPT-SS-PA conjugate	CPT	Great association efficiency; high stability in neutral medium and simulated gastrointestinal fluids; great cytotoxicity to various cancer cell line; enhanced oral bioavailability	[286]
Nanostructured lipid carriers (NLCs)	Soy lecithin; glyceryl; tridecanoate; glyceryl tripalmitate; vitamin E acetate; Kolliphor HS15	Resveratrol	Showing stability in artificial gastric and/or intestinal fluids; significant anticancer activity against Hep-G2, human HCT-116, lymphoblastic leukemia cells (1301), and human MCF-7 cell lines; significant apoptotic properties; potent in vitro antioxidant activity	[294]
	Compritol; labrafil 1944; lecithin; Tween-80	ART	Small particle size; good stability; high entrapment efficiency; sustainably release; more cytotoxic than free ART; apoptosis of tumor cells	[295]
	Octyl decyl acid triglycerate; Tween-80; lecithin; Poloxamer 188; monostearin	Curcumin	Superior anti-cancer activity in inhibiting proliferation; inducing apoptosis of human HepG2 cells; increased total expression of DR5 protein; upregulated cell membrane expression of DR5	[296]
	Solid lipid (Cap MCM C10); liquid lipid (Capmul PG8); Tween 80; PL-90G	Ganoderic acid	Compatible in hepatic nodules, hepatic, non-hepatic, antioxidant parameters, in a significant manner (p < 0.001); interferes with various cancer signaling protein	[297]
Microemulsion and nanoemulsion	R9 peptide; egg phosphatidylcholine; Mal-PEG-DSPE; PEG-DSPE; soya bean oil	Curcumin	Low cytotoxicity on HEC and low haemolytic activity; anti-inflammatory effect; increased accumulation of R9-CmLN in liver and lungs	[310]
Ethosomes and transfersomes	Folate-modified TPGS; PEG1000; cholesterol	Docetaxel	Low particles sizes and polydispersity index; high encapsulation efficiency; higher permeability into 3D U-87 MG spheroid; selectivity of transfersomes to tumoral cells	[269]
	HSPC; DSPE-PEG2000; cholesterol	Eugenol and cinnamaldehyde	Improved formulation stability and percutaneous drug absorption; increase interstitial cells of Cajal; excellent deformability; improved efficacy against UC	[324]
Polymer micelles (PMs)	PEGylated PLGA polymer (Resomer® RGPd50105 and RGPd5055;	Digoxin	Across BeWo b30 cell monolayers easily; high encapsulation efficiency and sustained release; increased the permeability of digoxin	[418]
	Chondroitin sulfate;	Docetaxel	High permeability and cytotoxicity of Cys-DTX prodrug, targeting transportation of encapsulated redox-responsive Cys-DTX prodrug; improved permeability in tumor tissues, enhanced cytotoxicity and decreased side effects	[398]
	Cystamine; Xanthan gum	Resveratrol	Good redox responsiveness; biocompatible; controlled in vitro drug release similar to the internal environment of tumor cells	[333]
	PEG-40 hydrogenated castor oil	Curcuminoid	Physically stable for at least two months; uniform droplets size and low polydispersity	[419]
Polymeric vesicles	(Poly (ethylene glycol)-*b*-poly (propylene thioether) (PEG-*b*-PPS))	Hydroxychloroquine (HCQ); tunicamycin (Tuni)	Simultaneously inducing endoplasmic reticulum (ER) stress and autophagic flux blockade; inhibiting tumor metastasis	[344]
	PEG-PLA/PEG-PBD hybrid vesicles	Paclitaxel	Thick hydrophobic membrane and an aqueous lumen to efficiently carry both hydrophobic and hydrophilic drugs; higher maximum tolerated dose; controlled drug release; two-fold higher cell death in tumors than free drug	[345]
Polymer hydrogels	Poly(N-isopropylacrylamide) and PEG	Artemisinin	Maintain the drug concentration at a therapeutic level for up to 10 days	[351]
	Poloxamer and chitosan	Herbal formula	Sustained release and enhanced nasal absorption of the active compounds	[353]
Gold nanoparticles (Au NPs)	DO powder; AuNPs	Dendrobium officinale (DO)	Better anti-tumor efficiency compared with DO extraction alone without increasing toxicity in vivo and in vitro	[362]
	AuNPs	Licochalcone A	Increased solubility in aqueous solution; green method	[363, 364]
	AuNPs	Pholiota adiposa	Significantly improved immune regulation and anti-tumor effect in comparison; no toxicity both in vivo and in vitro	[365]
	AuNPs	Paeonia mountan	Fulfills the requirement of ideal nanodrug and it potentially inhibited the inflammation in in vitro murine microglial BV2; alleviates the neuroinflammation and improves the motor coordination in Parkinson induced mice	[366]
Mesoporous silica nanoparticles (MSNs)	Pluronic F127; CTAB; TWEEN20; MSNs	Resveratrol	Solubility, drug release, and transport enhancement of resveratrol; enhanced anti-inflammatory activity	[372]
	Lipid-coated MSN@p(NIPAM-*co*-MA)	EVO; BBR	Improved efficacy and biocompatibility of the drug pair; desirable drug profiles at the low pH and higher temperature of the tumor microenvironment; excellent synergistic therapy effects in vitro and in vivo; lower systemic toxicity	[374]
	Hyaluronan (HA) or polyethyleneimine-FA (PEI-FA); MSNs	Curcumin	More precise targeting and higher accumulation in tumors; good biocompatibility and low toxicity; inhibited the tumor growth to a greater degree	[375]
	FA–conjugated mesoporous silica nanoparticles (FA-MSNs)	Rhodojaponin III	Prolonged RJ-III release in vitro; reduced the cytotoxicity of RJ-III (P < 0.01); good targeting effect; improved the LD50 value of RJ-III in mice	[376]
Organic/Inorganic Nanohybrids	Indocyanine green (ICG); perfluoropentane (PFP); MSNs; PEG	Paclitaxel	Effective intracellular ICG deliver; NIR-responsive hyperthermia; permitting photothermal therapy and photoacoustic imaging; potent and synergistic chemo-photothermal therapy	[382]
	PEG-*b*-P(CPTM-*co*-MPS) and PEG-*b*-P(CPTHM*-co*-MPS)	CPT	Codelivery both hydrophobic and hydrophilic drugs; site-specific synchronized corelease enhanced combination chemotherapeutic efficacy and reduced systemic toxicity	[384]
	N-(2-hydroxypropyl methyl) acrylamide (HPMA) copolymer-gadolinium-paclitaxel-Cyanine5.5 (pHPMA-Gd-PTX-Cy5.5)	Paclitaxel	Enhanced imaging capacity of the theranostic nanomedicine; residence time significantly prolonged; increased accumulation at the tumor site; inhibited proliferation and induced apoptosis of the 4T1 murine breast cancer cells	[420]
	Monomethoxy-poly(ethylene glycol)-*b*-poly(lactide) (mPEG-PLA)	Docetaxel	Clear spherical shape; sustained release of the drug; time-dependent anticancer effect in the squamous cancer cells; significantly higher cancer cell apoptosis in HSC-3 cancer cells; controlled the tumor progression in HSC-3 cancer cells	[400]
	Polyethylene glycol-BA (PEG-BA)	BA	Increased NFκB/p65 protein expression; comparable antioxidant potential with ascorbic acid; improved reduction of hydroperoxide levels	[399]
Nanocarriers based on active ingredients from TCMs	Baicalin and flavonoid glycosides	Baicalin and flavonoid glycosides	Significantly enhanced bacteriostatic activity; stronger affinity to bacteria; good biocompatibility; excellent antibacterial performance	[409]
	BBR and cinnamic acid	BBR and Cinnamic acid	better inhibitory effect on multidrug-resistant *S. aureus* (MRSA) and stronger ability for biofilm removal; nonhemolytic with little toxicity in vitro and in vivo	[412]
	Lipophilic alkyl BBR derivatives (BDs) and rhamnolipids (RHL)	BDs and RHL	Enhanced hydrophilicity, successfully penetrated through mucus layer without interacting with mucins; substantial ability to eradicate *H. pylori* biofilms; inhibited the adherence of H. pylori on both biotic and abiotic surfaces	[413]
	Ursolic acid	Ursolic acid	Near-spherical shape; higher antiproliferative activity; significantly caused apoptosis; decreased the expression of COX-2/VEGFR2/VEGFA; increased the immunostimulatory activity of TNF-α, IL-6, and IFN-β; inhibiting tumor growth and having the ability of liver protection in vivo	[408]
	*Poria cocos*; *Liquidambar formosana*	*Poria cocos*; *Liquidambar formosana;* PTX	Improved therapeutic effect on tumors through synergistic action; reducing chemotherapeutic cardiotoxicity and prolonging survival; superior anti-inflammatory efficacy	[409]
	Ursolic acid and PTX	Ursolic acid and PTX	Facile structure regulation and drug loading; synergistic therapeutic efficacy; reducing the risk of liver damage; benefiting and eliminating the trouble of the toxic side effects	[414]

## Challenges and regulatory considerations

TCMs faces several challenges in clinical practice, including poor bioavailability, low solubility, short in vivo release time, and certain side effects. This review summarizes some of the TCMs commonly found in this field, including terpenoids, polyphenols, flavonoids, alkaloids, and quinones. Researchers have made significant efforts to address these issues, and nanocarriers have received considerable attention due to their ability to improve the solubility of TCMs, excellent stability, enhanced absorption capacity, and improved sustained release effect and targeting ability. In addition, this review consolidates the primary nanocarriers employed in this area, such as liposomes, nanostructured lipid carriers, solid lipid nanoparticles, microemulsions, polymer nanocarriers, inorganic nanocarriers, hybrid nanocarriers and prodrug-based nanocarriers. These nanocarriers have displayed enormous potential in enhancing the effectiveness of TCMs and resolving the obstacles related to their clinical usage.

Despite numerous reports in the literature, the development of nanomedicine for TCMs delivery remains at a nascent stage. The use of TCMs in nanomedicine is currently limited due to a narrow scope of application, insufficient theoretical knowledge support, and incomplete basic research. Uncertainty about the specific efficacy of TCMs components against particular diseases further complicates the development of effective nanocarriers. Furthermore, implementing nanocarriers into nanomedicine may result in possible elevated toxicity and immunogenicity, along with additional manufacturing expenses and unpredicted hazards. Thus, the development of new nanocarriers requires conclusive evidence demonstrating the clinical efficacy, stability, and cost-effectiveness of certain nanocarriers. Attaining a more profound comprehension of metabolic pathways is crucial to guarantee the safety of nanocarriers in clinical applications and offer guidance for designing such carriers logically. To advance this field, broad pharmacokinetic and pharmacodynamic investigations of nanocarriers loaded with fractions of TCMs are necessary. By answering key questions about drug delivery mechanisms, pharmacokinetic properties, safety, and efficacy, these studies will guide the rational design and development of effective nanocarriers for TCMs delivery.

Guided by TCMs theory, nanotechnology is poised to become an integral part of TCMs modernization development. The integration of nanotechnology and TCMs represents a promising frontier in the modernization of traditional medicine, with the potential to revolutionize the field and transform healthcare as we know it. The cycle of clinical conversion is long, mainly considering safety, effectiveness, and production costs. With the increasing emphasis on basic research, the continuous innovation of delivery systems and manufacturing technologies, the gradual improvement of guiding norms, the further standardization of clinical research, and the rapid development of supporting technologies such as artificial intelligence and single cell sequencing, we have reason to believe that TCM-based nanomedicines will soon move towards clinical applications.

## Data Availability

The data that support the findings of this study are available from the authors upon reasonable request.
